# Spatial transcriptomics reveals a molecular tumor budding signature in head and neck cancer

**DOI:** 10.1186/s13073-026-01612-2

**Published:** 2026-04-15

**Authors:** Iordanis Ourailidis, Markus Ball, Vanessa Vogel, Min He, Hairong Wang, Stella Beomseo Kim, Sarah Böning, Barbara Wollenberg, Klaus Dietrich Wolff, Katja Steiger, Carolin Mogler, Anette Duensing, Stefan Duensing, Peter Schirmacher, Albrecht Stenzinger, Olivier Gires, Daniel Kazdal, Martina Kirchner, Fabian Stögbauer, Melanie Boxberg, Jan Budczies

**Affiliations:** 1 Institute of Pathology, University Hospital Heidelberg, Heidelberg, Germany; 2 Faculty of Biosciences, Heidelberg University, Heidelberg, Germany; 3 Clinic and Polyclinic for Otorhinolaryngology, Ludwig Maximilian University of Munich, Munich, Germany; 4 Molecular Urooncology, Department of Urology, University Hospital Heidelberg, Heidelberg, Germany; 5 Department of Otolaryngology Head and Neck Surgery, School of Medicine, Technical University of Munich, Munich, Germany; 6 Department of Oral and Maxillofacial Surgery, Technical University of Munich, Munich, Germany; 7 School of Medicine and Health, Institute of General and Surgical Pathology, Technical University of Munich, Munich, Germany; 8 Comparative Experimental Pathology, School of Medicine, Technical University of Munich, Munich, Germany; 9 Precision Oncology of Urological Malignancies, Department of Urology, University Hospital Heidelberg, Heidelberg, Germany; 10 Pathologie Starnberg MVZ GmbH, Starnberg, Germany; 11 Center for Personalized Medicine (ZPM), Heidelberg, Germany

**Keywords:** Tumor budding, Head and neck squamous cell carcinoma, Spatially resolved transcriptomics, Partial epithelial-mesenchymal transition, Molecular biomarker, MEK inhibition

## Abstract

**Background:**

Tumor budding (TB) is a histopathological feature associated with poor prognosis across multiple cancer types, including head and neck squamous cell carcinoma (HNSCC). Tumor buds represent the earliest traceable local invasion and are considered the before origin of minimal residual disease, local recurrence, and metastasis. However, the molecular processes underlying this phenomenon in HNSCC remain incompletely characterized, particularly with regard to intratumoral gene expression heterogeneity.

**Methods:**

We performed whole-transcriptome spatial transcriptomics on tissue sections from Human Papillomavirus (HPV) negative HNSCCs, sampling distinct regions of interest encompassing tumor buds, tumor bulk from budding and non-budding tumors, and adjacent stroma. Differential gene expression analyses led to the development of a 28-gene tumor budding signature (TBS) that separated tumor buds from all other tissue types. The TBS was validated using bulk RNA-seq (TCGA-HNSC), single-cell RNA-seq, and independent spatial transcriptomics datasets. Associations of the TBS with responses to drugs were evaluated in pharmacogenomic datasets and findings were further investigated in a 3D invasion model including MEK inhibition.

**Results:**

Tumor buds exhibited distinct transcriptional programs with upregulation of epithelial-mesenchymal transition markers and extracellular matrix remodeling genes. The TBS effectively identified tumor buds in the spatial transcriptomics dataset (AUC = 0.97), separated budding and non-budding tumors in the bulk RNA-seq TCGA-HNSC dataset (AUC = 0.8), and predicted overall survival in the latter dataset (HR = 1.54, *p* = 0.02). Analyses of single-cell and spatial transcriptomics datasets confirmed TBS expression primarily in malignant cells, its association with a hybrid epithelial-mesenchymal state, its expression predominantly at the leading edges of tumors, and its induction via subtypes of epidermal growth factor receptor activities. Pharmacogenomic analysis revealed that TBS-high squamous cell carcinoma cell lines were sensitive to MEK inhibitors, a finding validated in a 3D model of early local invasion.

**Conclusions:**

Integrated spatial-molecular profiling established a molecular bud biomarker facilitating the quantification of TB in both tumor tissues and cultured cells. Insights from the analysis of diverse datasets contribute to a better understanding of the molecular mechanisms underlying tumor invasion, improved risk stratification, and the development of new therapeutic approaches in HNSCC.

**Supplementary Information:**

The online version contains supplementary material available at 10.1186/s13073-026-01612-2.

## Background

Head and neck squamous cell carcinoma (HNSCC) is a major cause of cancer-related morbidity and mortality worldwide [1]. Despite multimodal treatment strategies and the addition of immunotherapy to the treatment arsenal, the prognosis of HNSCC remains poor. Besides the detection of active human papillomaviruses (HPV) in oropharyngeal tumors, not a single molecular marker has entered routine diagnostics, hence the urgent need for better predictive and prognostic tools [2]. Tumor invasion as well as local and distant metastasis formation are critical processes in the progression of HNSCC. A key histopathological feature associated with these processes is tumor budding (TB), defined as the presence of single cells or small clusters of up to four cancer cells detached from the main tumor bulk [3, 4]. TB has been linked to aggressive disease behavior and poor prognosis in HNSCC and is correlated with epithelial-mesenchymal transition (EMT), a process crucial for cancer cell migration, metastasis, and treatment resistance [5]. While it serves as an independent prognostic factor in various cancers [6–8], including HNSCC [4, 9–11], the underlying molecular mechanisms of TB remain incompletely understood [5].

Recently, we have analyzed the molecular basis of TB in HNSCC using comprehensive multi-omic approaches [12]. However, these analyses were based on bulk tissues and were unable to address tumor heterogeneity separating tumor cells of buds from the tumor bulk and other components of the tumor microenvironment (TME). Complementary, but missing spatial resolution, HNSCC has been investigated using single-cell RNA sequencing (scRNA-seq), revealing pronounced molecular heterogeneity predominantly in malignant cells [13–15]. Spatial transcriptomics has emerged as a powerful tool for studying cancer biology, including tumor heterogeneity, TME interactions, metastasis, and therapeutic resistance [16]. In this study, we leveraged digital spatial profiling (DSP) interrogating the whole transcriptome atlas (WTA) to characterize gene expression programs associated with TB by comparing budding with non-budding tumor regions and analyzing both the tumor and surrounding stromal compartments. We developed a molecular tumor bud biomarker and validated and further analyzed it in external datasets of bulk RNA-seq, scRNA-seq, and spatial transcriptomics data. Finally, by relating the tumor bud biomarker to drug response data of cultured cells and studying a 3D invasion model, we explored therapeutic vulnerabilities associated with TB. 

## Methods

### Study cohorts and datasets

This study integrates data from both in-house spatial and publicly available cohorts and datasets. An overview of all cohorts and the analyses performed on each is provided below; detailed descriptions of sample acquisition, processing, and quality control and analytical methods are provided in the respective subsections that follow.

The in-house spatial transcriptomics cohort comprised formalin-fixed paraffin-embedded (FFPE) tissue sections from 43 immunohistochemically p16-negative HNSCC patients (24 budding, 19 non-budding) collected at the Technical University of Munich. Spatial transcriptomics was performed using the NanoString GeoMx DSP platform with the WTA probe set. This cohort was used for differential gene expression analysis, tumor budding signature (TBS) development, pathway activity and regulatory network analyses, differential correlation analysis, and epithelial-mesenchymal phenotype characterization. The clinicopathological details of this cohort are provided in the *Sample collection and patient cohort* subsection, Additional File 1: Table S1, and Additional File 1: Fig. S1. The data have been deposited in NCBI GEO under accession number GSE300414 [17].

The Cancer Genome Atlas (TCGA) TCGA-HNSC cohort (*n* = 292 HPV-negative HNSCC samples) provided bulk RNA-seq gene expression data and digitized H&E slides from the Genomic Data Commons portal and the TCGA PanCanAtlas publications [18]. Tumor budding scores were determined as previously described [10, 11]. This cohort was used for TBS validation and survival analyses. Case selection criteria and budding scores are detailed in the *TCGA-HNSC dataset* subsection and Additional File 1: Table S2.

Two scRNA-seq datasets were used for single-cell-level validation: (i) the Puram et al. 2017 dataset (GSE103322 [13] with 18 HNSCC primary tumors and 5 matching lymph node samples) was used for TBS validation, differential gene expression, pathway activity, and pEMT analyses and (ii) the Choi et al. 2023 dataset (GSE181919 [19] with 13 HPV-negative HNSCCs, 4 leukoplakia, and 6 matched normal samples) was used as a second validation cohort and for EGFR-activity subtype analysis. Processing details are described in the *Single-cell RNA sequencing datasets* and *EGFR activity subtype analyses* subsections.

The Arora et al. 2023 spatial transcriptomics dataset (GSE208253 [20] with 12 oral cavity SCCs profiled with 10x Genomics Visium) served as an independent spatial validation cohort to assess TBS expression across tumor core, transitory, and leading edge regions, as detailed in the *Spatial transcriptomics analysis of public dataset* subsection.

Pharmacogenomic data from the Dependency Map (DepMap) portal (release 24Q2 [21, 22]) were used to evaluate associations between TBS and drug sensitivity in a panel of 53 to 85 squamous cell carcinoma cell lines (depending on data availability per compound) from head and neck (HNSCC), lung (LUSC), and esophageal (ESCA) squamous cell carcinomas, as described in the *Drug responses and tumor budding signatur*e subsection.

FaDu cell line transcriptomics data (GSE200421 [23]) were used to assess TBS changes upon EGF stimulation at six and 72 h, complementing the functional 3D invasion experiments described in the *Cell lines and inhibitor viability test* and *3D local invasion model* subsections.

### Sample collection and patient cohort

Tumor sections from formalin-fixed, paraffin-embedded (FFPE) tissue of primary tumors of 43 immunohistochemically p16-negative HNSCC patients (24 exhibiting budding and 19 non-budding) derived from locations known to give rise to non-HPV-related HNSCC (therewith excluding oropharyngeal cancers) were mounted on 24 slides (Additional File 1: Table S1). A total of 17 (39.5%) patients were female and the median age was 66 years (interquartile range: 17 years). A total of 15 cases (34.9%) were located on the upper and lower jaws, 14 cases (32.6%) on the tongue, 12 cases (27.9%) on the floor of the mouth, and two cases (4.7%) on the oral parts of the lip or cheek. Regarding TNM-staging, two cases (4.7%) were classified as pT1, 15 cases (34.9%) as pT2, 12 cases (27.9%) as pT3, 14 cases as pT4, 18 cases as pN0 (41.9%) 16 cases (37.2%) as pN + and 9 cases (20.9%) as pNX. Six cases had a histological tumor grade of G1, 22 cases of G2, and 15 cases of G3 (34.9%). By selecting tumors this way, we made sure to exclusively analyze non-HPV-related HNSCC resulting in a homogeneous cohort. Tissue microarrays (TMAs) were constructed, incorporating selected tumor regions from two to three representative cases per array. The selected areas measured approximately 25–50 mm², providing sufficient tumor content for downstream analyses. Regions of interest were selected by IO and two board certified pathologists (MB, FS). Each slide included a tumor section from a budding case, and on 19 of the 24 slides, an additional section from a non-budding case was also included. Additional File 1: Fig. S1 and Additional File 1: Table S1 provide detailed information on the number and type of samples analyzed and a summary of clinicopathological data respectively. Analysis of the in-house spatial transcriptomics cohort generated for this study was conducted in accordance with the Declaration of Helsinki and authorized by the ethics commission of the University Hospital Rechts der Isar (vote 2023-543-S-SB). The spatial transcriptomics data generated in this study have been deposited in the NCBI Gene Expression Omnibus (GEO) under the accession number GSE300414 [17].

### Sample preparation

The sample preparation was performed as described in the GeoMx DSP Manual Slide Prep User Manual (following the RNA FFPE instructions, available from https://university.nanostring.com/geomx-dsp-manual-slide-preparation-user-manual, accessed on 04.12.2023). Five µm thick FFPE tissue sections were mounted on BOND Plus slides (Leica Biosystems, Nussloch, Germany). The slides were baked in a 60 °C drying oven for 75 min and were deparaffinized and rehydrated with three five-minute washes in CitriSolv (Thermo Fisher Scientific, Waltham, MA, USA), two five-minute washes in 100% EtOH (Carl Roth, Karlsruhe, Germany), one five-minute wash in 95% EtOH, and one one-minute wash in 1x phosphate-buffered saline (PBS; Sigma-Aldrich, Burlington, MA, USA). The slides were then dipped into diethyl pyrocarbonate (DEPC; Thermo Fisher Scientific, Waltham, MA, USA) treated water (at ~ 99 °C) for ten seconds and immediately transferred to a ~ 99 °C 1X Tris-ethylenediaminetetraacetic acid (Tris-EDTA; eBioscience, a part of Thermo Fisher Scientific, Waltham, MA, USA) jar and incubated for 15 min for target retrieval. After incubation, the slides were moved to room temperature 1x PBS and were washed for five minutes. The samples were then digested with one µg/mL Proteinase K (Thermo Fisher Scientific, Waltham, MA, USA) for 15 min at 37 °C and washed with 1x PBS for five minutes. The slides were then washed in 10% neutral buffered formalin (NBF; EMS Diasum, Hatfield, PA, USA) for five minutes, two times in NBF Stop Buffer for five minutes each time, and one time in 1x PBS for five minutes. The samples were then incubated overnight (16 h) at 37 °C with Buffer R (NanoString, now part of Bruker Spatial Biology, Billerica, MA, USA) and the WTA RNA detection probes (NanoString, now part of Bruker Spatial Biology, Billerica, MA, USA) in a light-non-permeable humid chamber, ensuring humid conditions by arranging DEPC-treated water-soaked Kimwipes on the bottom of the chamber. The reagent concentrations were adjusted for each run, where 200 µL x *n* Buffer R, 25 µL x *n* WTA Probe Mix, and 25 µL x *n* DEPC-treated water were used for the final solution, with *n* referring to the number of slides analyzed per run. 200 µL of the final solution was applied on each slide and HybriSlip Hybridization Covers (Grace Bio-Labs, Bend, OR, USA) covered the tissue during incubation. To remove off-target probes, two 25-minute stringent washes were performed in 50% formamide in 2x saline-sodium citrate (SSC; Sigma-Aldrich, Burlington, MA, USA) at 37 °C. The slides were then washed twice with 2x SSC for two minutes each time. For the morphology marker staining, the slides were placed in the light-non-permeable humidity chamber, covered with 200 µL Buffer W (NanoString, now part of Bruker Spatial Biology, Billerica, MA, USA) each at room temperature for 30 min. Then the Buffer W was removed and up to 200 µL of morphology marker solution [22 µL x *n* Syto 13 (NanoString, now part of Bruker Spatial Biology, Billerica, MA, USA), 5.5 µL x *n* PanCK (Clone: Ae1 + AE3; NanoString, now part of Bruker Spatial Biology, Billerica, MA, USA), 5.5 µL x *n* CD45 (Clone: 2B11 + PD7/26; NanoString, now part of Bruker Spatial Biology, Billerica, MA, USA), filling in with Buffer W to reach 220 µL x *n* final volume, where *n* is the number of slides per run] was applied on each slide in the humidity chamber. The slides were stained for one hour in the humidity chamber at room temperature. After staining, the solution was removed and the slides were washed in 2x SSC two times for five minutes each time and loaded on the GeoMx DSP instrument (NanoString, now part of Bruker Spatial Biology, Billerica, MA, USA).

### GeoMx DSP sampling

Slides were imaged in three fluorescence channels (FITC/525 nm, Cy3/568 nm, and Texas Red/615 nm) to visualize morphology markers, and regions of interest were segmented based on the expression of the morphology markers. For each tumor, two regions of interest (ROIs) were sampled from the bulk of the tumor and the surrounding stromal tissue. In budding cases, an additional two to three ROIs were sampled, focusing specifically on tumor bud cells and the adjacent stroma. Tumor buds were defined as single tumor cells or clusters of up to four tumor cells detached from the main tumor mass, as recommended in current TB guidelines for colorectal cancer. The remaining invasive regions of the main tumor mass, including clusters of five or more tumor cells (poorly differentiated clusters, PDCs), and the contiguous invasive tumor nests were classified as tumor bulk. Tumor bulk regions were selected from areas without visible budding activity and excluded the tumor buds themselves. Each ROI was then divided in distinct areas of illumination (AOIs) based on the morphology markers PanCK and CD45. PanCK-negative/CD45-positive cells were assigned the “immune” AOI, PanCK-negative/CD45-negative cells were assigned the “stroma” AOI, and PanCK-positive/CD45-negative cells were assigned the “tumor” AOI. Additional File 1: Fig. S1 provides detailed information on the number of samples and ROIs/AOIs per case. Each AOI was sequentially illuminated with UV and the UV-cleavable released tags were collected into 96-well plates. Importantly, the illumination order impacts tag availability, as tags released in the first AOI are not available for subsequent AOIs and tags released in the second AOI are not available for the third illumination round. To minimize off-target contamination, all samples were processed in a standardized illumination order, beginning with the “immune” AOI, followed by the “stroma” AOI, and finally the “tumor” AOI. This approach was designed to reduce the presence of immune and stromal off-target signals in the tumor AOI. Additionally, to ensure maximal inclusion of the rare tumor bud cells, we typically lowered the PanCK positive detection threshold and increased the CD45 positive detection threshold, with the thresholds empirically optimized for each ROI followed by re-evaluation of the tumor tissue segmentation and inspection of tumor buds inclusion in the tumor AOI. The lowering of the PanCK threshold was used to avoid excluding weakly stained tumor buds, while the increasing of the CD45 threshold was applied to prevent misclassifying tumor cells with low levels of CD45 signal as non-tumor. As a result, some CD45⁺ immune cells with lower expression levels may have been excluded from the immune compartment and included in the stroma compartment. This is supported by *post-hoc* analysis of *CD45* gene expression, which showed low *CD45* transcript levels in the tumor compartment, high levels in the immune compartment, and low to intermediate expression in stroma (Additional File 1: Fig. S2a-b). While this approach significantly improved the accuracy and fidelity of tumor bud sampling, it may have introduced immune cell contamination into stromal regions, which should be considered when interpreting stroma-specific gene expression results. Finally, keratin expression (*KRT5*, *KRT6A*, *KRT17*) was almost exclusively observed in the tumor compartment, with low to no expression in the immune and stroma compartments (Additional File 1: Fig. S2c-h).

### Library preparation and sequencing

The library preparation was performed according to the GeoMx DSP NGS Readout User Manual (available from https://university.nanostring.com/geomx-dsp-ngs-readout-user-manual, accessed on 04.12.2023). In short, the 96-well plate was dried by being left on the bench top overnight covered by a permeable membrane which was subsequently replaced by another permeable membrane and the plate was then spinned down. The samples were then rehydrated with ten µL nuclease-free water and the plate was pulse-centrifuged to 1000 x *g*. GeoMx Seq Code primers (NanoString, now part of Bruker Spatial Biology, Billerica, MA, USA) were used to amplify the WTA tags and add Illumina adaptor sequences and sample demultiplexing barcodes. The GeoMx Seq Code primer plate was thawed and the GeoMx Seq Code primer plate and the GeoMx NGS Master Mix (NanoString, now part of Bruker Spatial Biology, Billerica, MA, USA) were pulse-centrifuged to 1000 x *g*. The subsequent PCR reaction was set up on a PCR plate with two µL of GeoMx NGS Master Mix, four µL of GeoMx Seq Code primer mix (added to the corresponding PCR plate well), and four µL of DSP aspirate (added to the corresponding PCR plate well) used per well. The PCR plate was sealed with a PCR plate sticker, pulse centrifuged to 1000 x *g*, and incubated in the thermocycler with a 100 °C heated lid. The cycling conditions were as follows: UDG treatment at 37 °C for 30 min, followed by UDG deactivation at 50 °C for ten min. Initial denaturation was performed at 95 °C for three minutes. Amplification consisted of 18 cycles of denaturation at 95 °C for 15 s, annealing at 65 °C for 60 s, and extension at 68 °C for 30 s. A final extension was carried out at 68 °C for five minutes, followed by an indefinite hold at 4 °C. The PCR products were pooled using four µL of each PCR product (including the no-template control, NTC) and the remainder of the NTC PCR product was placed into a separate tube. The tubes with the sample pool and the NTC went through the same AMPure cleanup protocol using the 1.2-fold volume AMPure XP beads (Beckman Coulter, Brea, CA, USA) and the libraries were sequenced on an Illumina NextSeq 550 with 27 × 27 paired end reads.

### Bioinformatics analysis

BCL to FASTQ conversion was performed for each sequencing run separately using DRAGEN and the following command line options: dragen --bcl-conversion-only true --bcl-input-directory /path/to/BCL/directory --output-directory /path/to/FASTQ --sample-sheet /path/to/SeqCodeIndices/SeqCodeIndices.csv.

FASTQ files were processed for each sequencing run separately using the NanoString GeoMx NGS Pipeline v2.3.3.10 and the following command line options: geomxngspipeline --in=/path/to/FASTQ --out=/path/to/DCC --ini=/path/to/GNP_config.ini.

The following default options were used: --quality-trim-score = 20 (score used for quality trimming), --trim-adapter=true (enable/disable adapter trimming), --adapter-trim-match-length = 10 [number of adapter sequence characters that will be used for adapter matching and trimming (starting from 3’ end)], --adapter-trim-max-mismatch = 3 (max number of character mismatches allowed during the adapter matching and trimming), --stitching-max-mismatch = 2 (max base pair mismatches allowed during stitching. Reads that exceed this value will be dropped), --stitch-shift=true (apply + 1/-1 sequence shift to find the best reads alignment for stitching), --barcode-max-mismatch = 1 [max number of mismatches allowed during the matching to the barcodes from the ini file. Reads that exceed this value will be dropped (not matched)], --dedup-hd = 1 [hamming distance used for the unique molecular identifier (UMI) deduplication]. For all downstream analysis, the deduplicated read counts were used. For each segment, additional quality control (QC) thresholds were applied to ensure data reliability. The minimum number of reads per segment was set at 40,000, the minimum number of successfully stitched reads at 80%, and the minimum number of aligned reads at 70%. Additionally, sequencing saturation was required to be at least 50%, while non-template control (NTC) wells were limited to a maximum of 1,000 counts. Each segment (that is a specific ROI-AOI combination) contained a set of negative probes; the geometric mean of the counts of the negative probes was calculated per segment and used to define the limit of quantification (LOQ), according to the formula *LOQ* = max (2, NegGeoMean × NegGeoSD^2^), where NegGeoMean is the geometric mean of the counts of the negative probes and NegGeoSD is the geometric standard deviation of the counts of the negative probes for a given segment. Essentially, the LOQ is defined as 2 standard deviations above the negative control mean and at minimum as at least 2 counts. Using the segment-specific LOQ thresholds, segments with exceptionally low signal (fewer than 5% of the genes detected above LOQ) and genes with low detection rates (fewer than 10% of the segments having detected a given gene above LOQ) were filtered out (Additional File 1: Fig. S1). The final post-QC dataset consisted of 239 segments and 7,795 genes. All segment-level QC thresholds and LOQ filtering were performed on raw probe-level counts prior to normalization. The raw counts of the post-QC dataset were normalized to upper quartile (UQ or Q3) normalized counts. A Uniform Manifold Approximation and Projection (UMAP) analysis of the normalized expression profiles of the samples was performed to identify heavy outliers and/or batch effects originating from the sequencing run (Additional File 1: Fig. S1f); the analysis shows no evidence of heavy outliers or strong sequencing-run-driven clustering. Last, for each case, the mean of the normalized counts for each gene was calculated for segments belonging to the same segment type.

### Spatial transcriptomics differential gene expression analysis and tumor bud biomarker development

Within-tumor (intratumoral) differential gene expression analysis (DGEA) was performed by comparing tumor bud ROIs to non-budding areas of the bulk of budding tumors (hereafter referred to as “budding”), while between-tumor (intertumoral) DGEA compared ROIs from the bulk of budding tumors to the bulk of non-budding tumors (hereafter referred to as “non-budding”). A paired Wilcoxon test was used for within-tumor comparisons, whereas an unpaired Wilcoxon test was used for between-tumor comparisons, separately analyzing the tumor and the tumor-adjacent stroma compartments, and adjusting for multiple testing using the Benjamini-Hochberg method, controlling false discovery rate (FDR) at 5%. DGEA of the immune compartment was not conducted due to the limited number of immune segments post-QC (Additional File 1: Fig. S1e). Additionally, to identify genes whose expression marks tumor buds in the tumor microenvironment, we performed a set of DGEA comparing tumor buds to all other segments and segment types, using either a paired Wilcoxon test or Wilcoxon test, as appropriate, and controlling FDR at 5%. The paired comparisons included buds (tumor) against stroma adjacent to buds, budding tumor bulk (as previously described), and stroma adjacent to budding tumor bulk, with each comparison as a separate analysis. The unpaired comparisons included buds (tumor) against non-budding tumor bulk, stroma adjacent to non-budding tumor bulk, and the immune compartment (pooled budding and non-budding immune segments), with each comparison as a separate analysis.

From the results of the DGEA, lists of differentially expressed genes (DEGs) were generated and only genes with an absolute fold change above 1.5 (at FDR 5%) were considered differentially expressed. The intersection of all these gene sets included 30 genes, 28 of which were overexpressed in the tumor buds. Based on these results, we calculated the tumor budding signature (TBS), defined as the mean over the expression of the 28 overexpressed genes, $\:TBS={\sum\:}_{i=1}^{28}lo{g}_{2}{x}_{i}$ wherein $\:{x}_{i}$ is the normalized expression values of gene $\:i$​. Additionally, a heatmap (clustering method: average, clustering distance: Manhattan) was generated to visualize the expression patterns of the genes comprising the TBS across all samples and compartments.

### TCGA-HNSC dataset

Digitized slides from The Cancer Genome Atlas head and neck cancer cohort (TCGA-HNSC) were downloaded from the Genomic Data Commons portal (https://portal.gdc.cancer.gov). Cases were selected according to the criteria established in Stögbauer et al.. 2023 [10] and Ourailidis et al.. 2025 [12] and for the selected cases the tumor budding score was determined as described in detail in previous publications [10, 11]. In short, tumor buds were defined as single cells or small clusters of up to four tumor cells detaching from the main tumor mass. Tumor budding was assessed on digitized HE-stained slides by counting the number of buds in ten consecutive high-power fields (HPFs; each 97,464 μm², corresponding to a 0.35 mm field diameter at 400× magnification), starting from the area with the highest number of tumor buds. Evaluation was performed by two experienced pathologists (FS and MB), with consensus reached in case of disagreement. Additional File 1: Table S2 provides the list of TCGA-HNSC samples used and their corresponding tumor budding scores. Cases were classified as “non-budding” when *TB* = 0 and “budding” when *TB* > 0 (with “low-budding” being cases with 0 < *TB* < 6 and “high-budding” cases when *TB* ≥ 6).

TCGA-HNSC normalized gene expression counts were downloaded from the TCGA - PanCanAtlas Publications [18] (file: https://api.gdc.cancer.gov/data/3586c0da-64d0-4b74-a449-5ff4d9136611, accessed on 06.10.2024). Each sample’s HPV status was inferred using the raw and normalized number of RNA-seq reads mapped to the HPV genomes, as described in Ourailidis et al. 2025 [12], and only HPV-negative samples for which a tumor budding score could be reliably determined were included in the analysis (*n* = 292). For each sample, the TBS was calculated as described in the tumor budding signature formula above.

### Single-cell RNA sequencing datasets

Single-cell RNAseq (scRNA-seq) data from 18 HNSCC cases and 5 matching lymph node samples were downloaded from the GEO repository GSE103322 [13]. Using the scRNA-seq gene expression values, we calculated the TBS (as described above) for each cell. The differences in the TBS between the cancer cells (primary tumor) and each other cell type were assessed with a Wilcoxon’s test, while the differences in the TBS of the cancer cells of each patient were assessed with a Kruskal-Wallis test. On top of calculating each cell’s TBS, each cell received a classification (“TBS-high” and “TBS-low”) based on the TBS cutoff calculated using CutoffFinder [24] and the distribution of TBS values of the cancer-cells of the primary tumors, which exhibited a bimodal distribution. Then, a set of DGEA was conducted comparing the TBS-high and TBS-low cancer cells of the primary tumors for the whole dataset and for each patient individually using Wilcoxon’s test and controlling FDR at 5%. Only patients with at least 20 TBS-high and 20 TBS-low cells were included in the DGEA. The overlap between the spatial transcriptomics DEGs and the scRNA-seq DEGs was assessed using a Fisher’s test and visualized with a Venn diagram and the genes comprising the TBS were excluded from the gene universe. Last, using the top 8,000 genes ranked by the standard variation of their expression, a set of tSNE plots were produced, each point representing a cell annotated based on the TBS classification and the cell type or patient it is assigned to.

Additionally, we used the HNSCC scRNA-seq dataset retrieved from the GEO repository GSE181919 [19] as a second scRNA-seq validation dataset. Raw UMI count matrices for HNSCC samples were processed with Seurat (v5.3.1) [25]. Cells with fewer than 200 detected genes, more than 8,000 detected genes, or > 10% of total UMI counts derived from mitochondrial genes were excluded. Genes expressed in fewer than 0.1% of remaining cells were filtered out. UMI counts were normalized using Seurat’s LogNormalize method with a scale factor of 10,000, yielding log-normalized expression values that were used for all downstream analyses. TBS was calculated as the mean of the normalized gene expression values per cell. Similarly to the previously described scRNA-seq analysis and using the HPV-negative HNSCC (*n* = 13) and leukoplakia (*n* = 4) samples (and the corresponding normal samples when available, *n* = 6), differences in the TBS between the cancer cells and each other cell type were assessed with a Wilcoxon’s test, while differences in the TBS of the cancer cells of each patient were assessed with a Kruskal-Wallis test. TBS differences between the epithelial cells of the normal samples, the epithelial cells of the leukoplakia samples, and the cancer cells of the primary tumors were assessed with Wilcoxon’s tests. Each cell received a classification (“TBS-high” and “TBS-low”) based on the TBS cutoff calculated using CutoffFinder [24] and the distribution of TBS values of the cancer-cells of the primary tumors. Then, a DGEA was conducted comparing the TBS-high and TBS-low cancer cells of the primary tumors using Wilcoxon’s test and controlling FDR at 5%. The overlap between the GSE103322 scRNA-seq DEGs and the GSE181919 scRNA-seq DEGs was assessed using a Fisher’s test and visualized with a Venn diagram and the genes comprising the TBS were excluded from the gene universe.

### EGFR activity subtype analyses

Epidermal growth factor receptor (EGFR) activity subtypes were analyzed in scRNA-seq data from the GEO repository GSE181919 [19], as described in Zhou et al. [26]. Briefly, non-negative matrix factorization (NMF) was used to cluster epithelial cells according to their EGFR activity using the “REACTOME_SIGNALING_BY_EGFR” gene set from the GSEA MSigDB (https://www.gsea-msigdb.org/gsea/msigdb/human/geneset/REACTOME_SIGNALING_BY_EGFR.html). EGFR activity subtypes were defined by their most differential genes and were represented as uniform manifold approximation and projection (uMAP) plots. EGFR (as above) and EMT (https://www.gsea-msigdb.org/gsea/msigdb/human/geneset/HALLMARK_EPITHELIAL_MESENCHYMAL_TRANSITION.html) activities, and TBS scores were inferred for all distinct EGFR-activity subtypes using *AddModuleScore*.

### Spatial transcriptomics analysis of public dataset

The spatial transcriptomics dataset GSE208253, comprising 12 oral cavity SCC samples profiled using the 10x Genomics Visium platform, was retrieved from the GEO repository. Preprocessing steps and data normalization of the dataset were described earlier [26]. Unsupervised Louvain clustering served to define cluster 1 as “Tumor Core” (TC; *CLDN4* as marker), cluster 3 as “Leading Edge” (LE; *LAMC2* as marker), and cluster 2, exhibiting features of both, as “Transitory region”, as described by Arora et al. [20]. Cluster annotation was used to define spots corresponding to the LE, transitory, and TC regions from each sample. To detect expression differences of the TBS between all tumor clusters, differential analysis was conducted using a one-way ANOVA test to assess the statistical significance, and a Tukey’s post-hoc test was then applied to identify specific pairs of clusters with significant differences (*p < 0.05, ****p < 0.01).

### Enrichment analysis

Enrichment analyses were performed using the cancer hallmark catalog of the Molecular Signatures Database (MSigDB) v7.5 [27] and the DEGs identified in the spatial transcriptomics and scRNA-seq datasets, as well as the TBS genes. For each gene set, an enrichment fold change of the percentages score was calculated using the formula $\:FC=\left(\frac{k}{K}\right)/\left(\frac{n}{N}\right)$, in which *k* refers to the number of genes in the MSigDB gene set, *K* refers to the number of DEGs, *n* refers to the number of common genes between the *k* and *K* sets, and *N* refers to the number of genes in the respective MSigDB catalog. The enrichment analysis was conducted twice for each analysis; once using the overexpressed DEGs and then using the underexpressed DEGs. The TBS contained only overexpressed DEGs. The enrichment of the functional categories was assessed using Fisher’s test. For the spatial transcriptomics, the enrichment results were visualized with barplots and for the scRNA-seq with a heatmap (clustering method: average, clustering distance: Manhattan).

### Pathway activity analysis

Using the normalized gene expression values of the in-house spatial transcriptomics cohort, signaling activity of 14 pathways was calculated with the Pathway RespOnsive GENes for activity inference (PROGENy) tool (R package progeny v1.28.0) [28] for each compartment of each segment type (progeny options: *organism* = “Human”, *scale* = T and *top* = 500). The PROGENy pathway activation scores of each compartment and segment type were compared using the Wilcoxon test and the Benjamini-Hochberg multiple testing correction was applied, controlling FDR at 5%. The PROGENy analysis was additionally performed using the scRNA-seq dataset (GSE103322) comparing the PROGENy pathway activation scores of the TBS-high and TBS-low primary cancer cells.

### Genetic regulatory network analysis

The genetic regulatory network analysis was based on the Algorithm for the Reconstruction of Accurate Cellular Networks (ARACNe)-inferred network of the TCGA-HNSCC cohort (R package aracne.networks v1.32) [29] containing 6,055 regulators, 19,722 targets and 423,104 interactions. The ARACNe TCGA-HNSCC network was subset to include interactions whose targets were in the DEGs (top 50 over- and top 50 underexpressed genes) between tumor buds and tumor bulk of budding tumors. GEne Network Inference with Ensemble of trees (R package GENIE3 v1.28.0) [30] with the normalized gene expression values of the genes in the final gene selection were used for weight calculation with a threshold of 0.0035 and Spearman correlation was used to calculate the correlation between each pair.

### Differential correlation of gene expression analysis

Differential correlation analysis was performed to identify changes in gene-gene correlations between the tumor buds and the bulk of the budding regions. Spearman correlation coefficients were calculated for all gene pairs in all compartment combinations (tumor-tumor, stroma-stroma, and tumor-stroma) of the tumor buds (and the adjacent stroma) and the bulk of the budding tumors (and the adjacent stroma) separately. Gene pairs with significant correlations (|Spearman rho| > 0.8 controlling FDR at 1%) in at least one of the conditions (buds or bulk of budding) were included in the downstream analysis. To assess statistical significance, we applied a Fisher’s Z-transformation and implemented a permutation test; sample labels (buds or bulk of budding) were randomly shuffled between the two conditions and correlation differences were recalculated over 100,000 permutations to generate a null distribution. Empirical *p*-values were derived by comparing observed differences in Z-values to this null distribution and multiple testing correction was applied controlling FDR at 1%. Genes with significantly altered correlations were additionally selected based on an absolute Z-value difference of at least 1.4. The resulting pairs of differentially correlating genes were visualized using gene networks, where nodes represent genes (distinguishing between stroma and tumor compartment gene expression) and edges represent significant correlation changes. To further annotate the transcription factor, ligand, and receptor genes in the differentially correlating gene pairs, annotated genes lists were downloaded from The Human Transcriptome database [31] (file: https://humantfs.ccbr.utoronto.ca/download/v_1.01/TF_names_v_1.01.txt, accessed on 30.10.2024) and the Cell-Cell Interaction Database [32] (files: https://baderlab.org/CellCellInteractions? action=AttachFile&do=get&target=ligands.txt and https://baderlab.org/CellCellInteractions? action=AttachFile&do=get&target=receptors.txt, accessed on 31.10.2024). Additionally, known gene-gene interactions were retrieved from the STRING [33] (files: https://stringdb-downloads.org/download/protein.links.v12.0/9606.protein.links.v12.0.txt.gz and https://stringdb-downloads.org/download/protein.info.v12.0/9606.protein.info.v12.0.txt.gz, accessed on 04.11.2024) and BIOGRID [34] (directory: https://downloads.thebiogrid.org/Download/BioGRID/Release-Archive/BIOGRID-4.4.239/BIOGRID-ORGANISM-4.4.239.tab3.zip, file: BIOGRID-ORGANISM-Homo_sapiens-4.4.239.Table 3.txt, accessed on 04.11.2024) databases and differentially correlating gene pairs with a known interaction were annotated. Last, genes in the differentially correlating gene pairs were annotated based on whether they were also differentially expressed between the bud and the bulk of budding conditions. Selected gene pairs were plotted using scatterplots to illustrate correlation differences between conditions.

### Receiver operating characteristic (ROC) analysis

To evaluate the discriminatory power of the TBS, we performed receiver operating characteristic (ROC) curve analyses. First, we assessed the ability of the TBS to distinguish tumor buds from all other sampled segments and compartments in the spatial transcriptomics dataset. Second, we applied the same analysis to the TCGA-HNSC cohort, using the TBS to differentiate budding from non-budding tumors. The area under the curve (AUC) was calculated to quantify the classification performance in both datasets and a Wilcoxon’s test was used to compare the two resulting groups.

### Epithelial-mesenchymal gene expression analysis and pEMT-TBS correlation

To assess the prevalence of epithelial and mesenchymal phenotypes, we curated a list of epithelial and mesenchymal/EMT-associated transcription factors and genes. Epithelial markers included Cadherin-1 (*CDH1*), Epithelial Cell Adhesion Molecule (*EPCAM*), Occludin (*OCLN*), Claudin 3 (*CLDN3*), Claudin 4 (*CLDN4*), Claudin 7 (*CLDN7*), Mucin 1, Cell Surface Associated (*MUC1*), Desmoplakin (*DSP*), Keratin 8 (*KRT8*), Keratin 18 (*KRT18*), Keratin 19 (*KRT19*), Keratin 7 (*KRT7*), Keratin 17 (*KRT17*), and Keratin 5 (*KRT5*). EMT/mesenchymal markers comprised Vimentin (*VIM*), Cadherin 2 (*CDH2*), Fibronectin 1 (*FN1*), Matrix Metallopeptidase 9 (*MMP9*), Matrix Metallopeptidase 14 (*MMP14*), Integrin Subunit Alpha 5 (*ITGA5*), Tenascin C (*TNC*), Serpin Family E Member 1 (*SERPINE1*), Integrin Subunit Beta 1 (*ITGB1*), Collagen Type I Alpha 1 Chain (*COL1A1*), Collagen Type I Alpha 2 Chain (*COL1A2*), Matrix Metallopeptidase 2 (*MMP2*), Periostin (*POSTN*), Fibroblast Activation Protein Alpha (*FAP*), Thy-1 Cell Surface Antigen (*THY1*), Secreted Protein Acidic And Rich In Cysteine (*SPARC*), Epithelial Membrane Protein 3 (*EMP3*), Podoplanin (*PDPN*), Collagen Triple Helix Repeat Containing 1 (*CTHRC1*), Thrombospondin 1 (*THBS1*), and Matrix Metallopeptidase 8 (*MMP8*). In addition, the transcription factors Snail Family Transcriptional Repressor 1 (*SNAI1*), Snail Family Transcriptional Repressor 2 (*SNAI2*), Zinc Finger E-Box Binding Homeobox 1 (*ZEB1*), Zinc Finger E-Box Binding Homeobox 2 (*ZEB2*), Twist Family BHLH Transcription Factor 1 (*TWIST1*), and Twist Family BHLH Transcription Factor 2 (*TWIST2*) were included. Gene expression levels for these genes were extracted from the spatial transcriptomics and scRNA-seq of Puram et al. 2017 [13] datasets. Heatmaps were generated (clustering method: average, clustering distance: Manhattan) to visualize the expression patterns of the marker genes across different sample groups (tumor buds, bulk tumor regions of budding tumors, bulk tumor regions of non-budding tumors for the spatial transcriptomics dataset, as well as TBS-high and TBS-low for the scRNA-seq dataset). The genes were additionally annotated based on whether they were differentially expressed in their respective datasets. Additionally, a partial EMT (pEMT) score was calculated for each tumor ROI/AOI (in-house spatial transcriptomics) and each primary tumor cell (scRNA-seq) using the top 15-genes associated with the pEMT program described in Puram et al. 2017 [13]. The pEMT score was calculated as the mean of the log2-transformed gene expression values of *SERPINE1*, Transforming Growth Factor Beta Induced (*TGFBI*), Matrix Metallopeptidase 10 (*MMP10*), Laminin Subunit Gamma 2 (*LAMC2*), Prolyl 4-Hydroxylase Subunit Alpha 2 (*P4HA2*), *PDPN*, *ITGA5*, Laminin Subunit Alpha 3 (*LAMA3*), Cadherin 13 (*CDH13*), *TNC*, *MMP2*, *EMP3*, Inhibin Subunit Beta A (*INHBA*), Laminin Subunit Beta 3 (*LAMB3*), and *VIM* and its correlation with the TBS was assessed using Spearman’s correlation.

### Survival analysis

To assess the prognostic value of the TBS, we performed survival analyses using the TCGA-HNSC cohort. Univariate and multivariate Cox proportional hazards models were used to evaluate the association between the TBS and overall survival (OS), adjusting for clinicopathological covariates including age, sex, tumor localization, American Joint Committee on Cancer (AJCC) stage, and tumor margin status. The hazard ratio (HR) and 95% confidence interval (CI) were calculated for each variable. Additionally, we optimized the TBS cutoff for optimal patient stratification based on survival outcomes using CutoffFinder [24] and visualized the survival differences using Kaplan-Meier curves. Statistical significance was determined using the log-rank test.

### Drug responses and tumor budding signature

To evaluate the relationship between the TBS and drug sensitivity, we analyzed publicly available pharmacogenomic data from the Dependency Map (DepMap) portal [21]. We focused on squamous cell carcinoma cell lines, mainly head and neck squamous cell carcinoma (HNSCC), lung squamous cell carcinoma (LUSC), and esophageal squamous cell carcinoma (ESCA) cell lines.

The following datasets were downloaded from DepMap Public 24Q2 (accessed on 07-01-2025) [22]:

- Gene expression data: log_2_(TPM_GeneExpression_+1) values for all genes across cell lines https://depmap.org/portal/data_page/?tab=allData&releasename=DepMap+Public+24Q2&filename=OmicsExpressionAllGenesTPMLogp1Profile.csv.
- Cell line metadata: Information on cell line characteristics https://depmap.org/portal/data_page/?tab=allData&releasename=DepMap+Public+24Q2&filename=Model.csv and https://depmap.org/portal/data_page/?tab=allData&releasename=DepMap%20Public%2024Q2&filename=OmicsProfiles.csv.
- Drug response data: Log-fold change (LFC) values representing drug sensitivity from the PRISM Repurposing Public 24Q2 dataset https://depmap.org/portal/data_page/?tab=allData&releasename=PRISM%20Repurposing%20Public%2024Q2&filename=Repurposing_Public_24Q2_Extended_Primary_Data_Matrix.csv and the accompanying metadata https://depmap.org/portal/data_page/?tab=allData&releasename=PRISM%20Repurposing%20Public%2024Q2&filename=Repurposing_Public_24Q2_Extended_Primary_Compound_List.csv.

The TBS was computed for each cell line using the TBS formula described above. Spearman correlation analyses were performed to assess associations between the TBS and drug response and FDR was controlled at 5%. Additionally, TBS scores were calculated for the FaDu cell line with and without EGF stimulation after six and 72 h using data published in Schinke et al. [23] and differences in the TBS levels with and without EGF stimulation were assessed with t-tests.

### Cell lines and inhibitor viability test

HNSCC cell line FaDu was purchased from ATCC (Manassas, VA, USA), regularly mycoplasma-tested, and verified via short-tandem repeat analysis. Cells were passaged in Dulbecco’s Modified Eagle Medium (DMEM) supplemented with 10% Fetal Calf Serum (FCS), 1% pen/strep, 5% CO_2_ atmosphere at 37 °C. Treatment with epidermal growth factor (EGF; 9 nM, Gibco Fisher Scientific, Munich, Germany), cetuximab (10 µg/mL, Erbitux, Merck Serono, Darmstadt, Germany), and avutometinib (10 or 25 nM; Selleckchem, Munich, Germany) were conducted under serum-free conditions in monolayer cultures and in collagen-embedded spheroids.

For vitality testing, 2,500 FaDu cells per well were seeded into 96-well plates in complete medium and incubated for 24 h at 37 °C with 5% CO₂ to allow cell attachment. After 24 h, cells were treated with serial dilutions of the RAF/MEK dual inhibitor avutometinib ranging from 10 nM to 1 μm. Each concentration was tested in triplicate wells. Cells were then incubated for an additional 72 h under standard culture conditions. Following treatment, 10 µL of Water-Soluble Tetrazolium 8 (WST-8) reagent (10% v/v of the total volume) mixed with serum-free medium was added directly to each well and incubated at 37 °C for 2 h in the dark. Absorbance was measured at 450 nm using a microplate reader. The optical density (OD) values were normalized to untreated controls to calculate relative cell viability. The percentage of viable cells at each concentration was plotted using GraphPad Prism 9.

### 3D local invasion model

The spheroid-based model of local invasion into extracellular matrix has been described in detail in Zhou et al. [26]. FaDu cell spheroids (3,000 cells/well) were allowed to form for three days in BIOFLOAT ultra-low attachment 96-well round-bottom plates (faCellitate, Mannheim, Germany; 3000 cells/well) and were transferred into 35 mm glass bottom dishes (Ibidi, Munich, Germany) in 200 µL of serum-free medium containing type I collagen (Corning, Oak Park, Bedford, MA, USA; 1.7 mg/mL). After collagen polymerization, EGF treatment (9 nM) was performed in the absence or presence of inhibitors/compounds as specified. Dual RAF/MEK inhibitor avutometinib was used at 10 and 25 nM and supplied simultaneously with EGF. Images were taken using a Leica DMi8 microscope 5x/10x in the PH channel (Leica, Nussloch, Germany) after 72 h. Invasive area and distance were quantified as described [26].

### Software and data analysis tools

All analyses were run in bash v5.1.16(1) and R v4.4.2 [35], with the latter being used for the statistical analysis and graphics generation. The R packages pheatmap v1.0.12 [36] and ComplexHeatmap v2.22.0 [37] were used for heatmap generation, pROC v1.18.5 [38] for the ROC analysis, survminer v0.5.0 [39] and survival v3.8.3 [40] for the survival analyses (including the univariate and multivariate analyses), Rtsne v0.17 [41] for the tSNE plots, ggraph v2.2.1 [42] and igraph v2.0.3 [43] for the visualization of the networks, VennDiagram v1.7.3 [44] for the Venn diagram, ggbeeswarm v0.7.2 [45] for the beeswarm plots, ggrepel v0.9.6 [46], ggtext v0.1.2 [47], ggforce v0.5.0 [48], circlize v0.4.16 [49], gridExtra v2.3 [50], RColorBrewer v1.1.3 [51], ggpubr v0.6.0 [52], and ggplot2 v3.5.1 [53] for plotting, doParallel v1.0.17 [54] for task parallelization, and Seurat v5.3.1 [25], tidyverse v2.0.0 [55], tidyr v1.3.1 [56], data.table v1.17.0 [57], NanostringNCTools v1.14.0 [58], GeomxTools v3.10.0 [59], GeoMxWorkflows v3.10.0 [60], and reshape2 v1.4.4 [61] for data analysis.

## Results

### Molecular characterization of tumor budding using Spatial transcriptomics

**Fig. 1:**
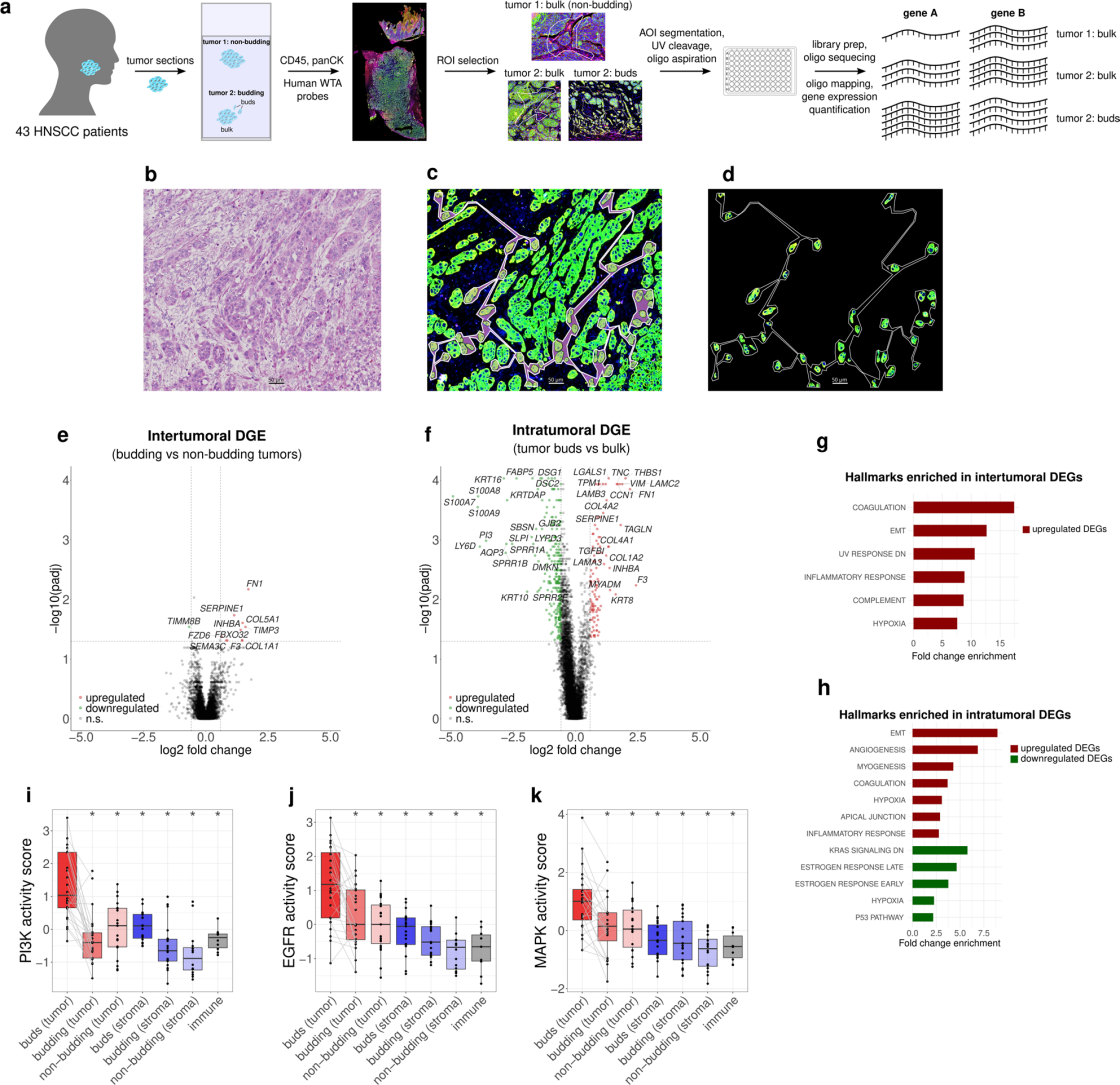
Molecular characterization of tumor buds in HNSCC. **a** Study overview. **b** Representative H&E-stained image of a selected ROI containing tumor buds with the sampled tumor buds outlined. **c** The same ROI visualized in the GeoMx DSP platform. **d** Segmentation of the tumor compartment of the ROI showing the cells selected for profiling. **e** Intertumoral and **f** intratumoral DGEA volcano plots. **g** Intertumoral and **h** intratumoral enrichment analyses using the MSiGDB HALLMARK gene sets and the differentially expressed genes. **i-k** PI3K, EGFR, and MAPK PROGENy pathway activation scores by compartment and segment type. Groups with significantly different distributions of PROGENy scores than the tumor buds PROGENy scores after multiple testing correction are marked with an asterisk

Spatial transcriptomics was performed on sections of 43 head and neck squamous cell carcinoma (HNSCC) patients selecting distinct regions of interest (ROIs) corresponding to tumor buds, tumor bulk from budding tumors, and tumor bulk from non-budding tumors and the corresponding adjacent stroma (Fig. 1a, Additional File 1: Fig. S1). Each ROI was segmented into tumor, stromal, and immune compartments using immunofluorescence markers, followed by compartment-specific barcode collection, and subsequent transcript quantification by next-generation sequencing (NGS). Figures 1b-d show a representative example of a tumor bud ROI with hematoxylin-eosin (H&E) and immunofluorescence staining and tumor bud selection before and after segmentation.

Differential gene expression analysis (DGEA) revealed distinct transcriptional profiles associated with tumor budding (TB) at both intertumoral (between a budding and a non-budding tumor, Fig. 1e) and intratumoral (between matched buds and bulk within a tumor, Fig. 1f) levels, with 11 and 379 differentially expressed genes (DEGs) respectively, which additionally suggests greater intratumoral than intertumoral transcriptional differences. In both comparisons, epithelial-mesenchymal transition (EMT) and coagulation genes including Fibronectin 1 (*FN1*), Coagulation Factor III (*F3*), and Serpin Family E Member 1 (*SERPINE1*) were among the most significantly upregulated genes. In the intratumoral analysis, the three subunits of laminin 332/laminin 5 (*LAMA3*, *LAMB3*, and *LAMC2*) were strongly overexpressed, highlighting their potential role in facilitating tumor cell detachment, invasion, and interaction with the extracellular matrix (ECM), together with a distinct expression pattern of keratins including overexpression of *KRT8* and underexpression of *KRT10* and *KRT16*. Other underexpressed genes in the tumor buds include the desmogleins *DSG1* and *DSC2*, two desmosomal cadherins that play essential roles in cell-cell adhesion and epithelial integrity.

Gene set enrichment analysis of the DEGs in the cancer hallmark catalog highlighted key biological pathways associated with TB: both intertumoral and intratumoral comparisons showed upregulation of EMT, coagulation, hypoxia, and inflammatory response pathways (Fig. 1g-h). Hence, TB is characterized by an EMT-like transcriptional program and ECM remodeling by malignant cells, suggesting gene expression alterations in tumor buds in response to the surrounding tumor microenvironment (TME). To further characterize signaling pathway activity across tumor segments and compartments, we applied PROGENy [28] to infer pathway scores from gene expression profiles. Hierarchical clustering based on pathway activity scores revealed distinct clustering of tumor buds (Additional File 1: Fig. S3), characterized by increased activity of the PI3K, EGFR, and MAPK pathways (Fig. 1i-k). These findings suggest a role of EGFR/MAPK/PI3K/Akt signaling in promoting and/or sustaining the budding phenotype representing potential vulnerabilities for therapeutic targeting.

Furthermore, we examined differential expression of genes in the stromal compartments. Additional File 1: Fig. S4a-f show representative H&E and immunofluorescence images of the segmentation of the stromal compartment. At the intertumoral level, comparing the stroma of budding versus non-budding tumors (Additional File 1: Fig. S4g), we observed an upregulation of *MMP14*, Inhibin Subunit Beta A (*INHBA*), and PDZ And LIM Domain 4 (*PDLIM4*), which play key roles in ECM remodeling, TGF-β signaling, and cytoskeletal organization. At the intratumoral level, comparing the stroma adjacent to tumor buds and bulk tumor stroma (Additional File 1: Fig. S4h), we found a notable downregulation of S100 protein genes and C-X-C Motif Chemokine Ligand 14 (*CXCL14*), suggesting a suppression of inflammatory signaling and altered immune-stromal interactions in the tumor bud-adjacent stroma. Our findings suggest that transcriptional changes in the stroma may contribute to the formation and/or persistence of tumor buds.

### Regulatory network and differential correlation of gene expression analyses reveal rewired relations in tumor budding

Analysis of HNSCC gene regulatory networks of the top intratumoral DEGs revealed substantial context-dependent rewiring in tumor buds compared to bulk tumor regions, involving altered connectivity for key hub genes (Additional File 1: Fig. S5). Junction Plakoglobin (JUP) was linked to the downregulation of epithelial and desmosomal genes such as *DSP*, *KRT6A*, *KRT16*, and *DSG3* in tumor buds. Caveolin 1 (*CAV1*) was linked to upregulated genes, specifically laminin-332 subunits *LAMA3*, *LAMB3, *and* LAMC2*. Notably, in tumor buds, *CAV1* acquired new regulators, namely *F3* and *PDPN*, which were additionally upregulated in tumor buds. Furthermore, Matrix Metallopeptidase 14 (*MMP14*) showed increased connectivity in the tumor buds’ regulatory network, regulating ECM proteins like Transforming Growth Factor Beta Induced (*TGFBI*) and Tenascin C (*TNC*).

**Fig. 2:**
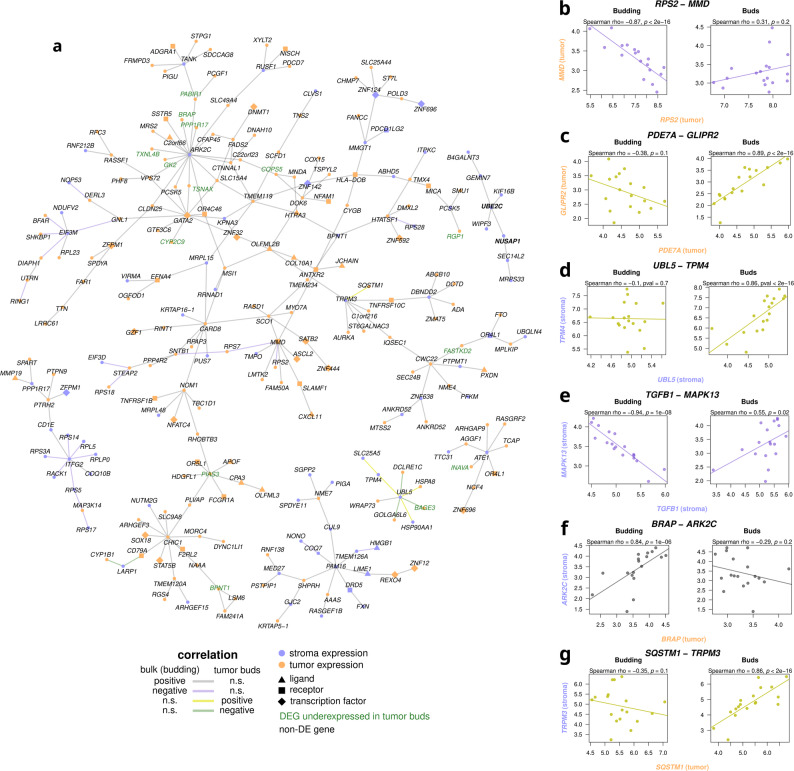
Differential correlation of gene expression between tumor buds and bulk. **a** Networks with more than 8 members. Each node refers to a gene, with tumoral (orange) and stromal (purple) expression shown separately. The shape of the node refers to a functional annotation (ligand, receptor, transcription factor) and gene pairs in bold font refer to known interactions. The edges connect two genes when their correlation of expression was differential, with different colors showing the direction of correlation change between tumor buds and bulk. **b-g** Selected gene pairs exhibiting differential correlation. The colors of the points and the x- and y-labels correspond to the colors of the network graph. The x and y axes refer to the Q3 normalized counts

Complementary differential correlation analysis between tumor buds and bulk demonstrated extensive rewiring of gene networks across intratumoral, intrastromal, and tumor-stroma compartments in tumor buds, with 292 intratumoral, 220 intrastromal, and 628 tumor-stroma differential correlations (Fig. 2a, Additional File 1: Fig. S6, Additional File 1: Fig. S7). Hub genes identified through this analysis included stroma-expressed genes, such as Arkadia (RNF111) C-Terminal Like Ring Finger Ubiquitin Ligase 2C (*ARK2C*), Arginyltransferase 1 (*ATE1*), Transient Receptor Potential Cation Channel Subfamily M Member 3 (*TRPM3*), and Ubiquitin Like 5 (*UBL5*), tumor-expressed genes, like Protein Inhibitor Of Activated STAT 3 (*PIAS3*) and Cysteine Rich Hydrophobic Domain 1 (*CHIC1*), and transcription factors, like Monocyte To Macrophage Differentiation Associated (*MMD*) and GATA Binding Protein 2 (*GATA2*). Within tumor cells, changes included loss of correlations, exemplified by a strong negative correlation between *RPS2* (ribosomal protein) and *MMD* (associated with proliferation) observed in the bulk of budding tumors, which was absent in tumor buds (Fig. 2a-b). Conversely, gain of correlations was noted, such as a strong positive correlation that emerged in tumor buds between *PDE7A* (linked to migration/invasion) and *GLIPR2* (promoting ERK1/2-mediated EMT), a correlation that was absent in bulk tumor (Additional File 1: Fig. S7, Fig. 2c). Intrastromal changes included gain of correlations, like the *TPM4* (regulator of the actin cytoskeleton implicated in ECM remodeling) and *UBL5* (Fig. 2d), as well as loss of correlations, like the gene pair Transforming Growth Factor Beta 1 (*TGFB1*) and Mitogen-Activated Protein Kinase 13 (*MAPK13*), both components of the MAPK pathway, which lost its positive correlation in tumor bud-adjacent stroma (Additional File 1: Fig. S6, Fig. 2e). Last, tumor-stroma crosstalk alterations were evident, as several correlations between tumor-expressed and stroma-expressed genes were either disrupted or gained in tumor buds. For instance, positive correlations between stromal *ARK2C* and several tumoral DEGs (like *BRAP*) were observed in the bulk of budding tumors but lost in the buds (Fig. 2f). Conversely, new correlations emerged, such as between stromal *TRPM3* and tumoral Sequestosome 1 (*SQSTM1*), which became strongly positively correlated in tumor buds (Fig. 2g). These findings highlight extensive rewiring of gene networks during TB, encompassing both the dissolution of existing co-expression patterns and the emergence of new correlations.

### A tumor budding signature derived from gene expression data identifies tumor buds and predicts survival

**Fig. 3:**
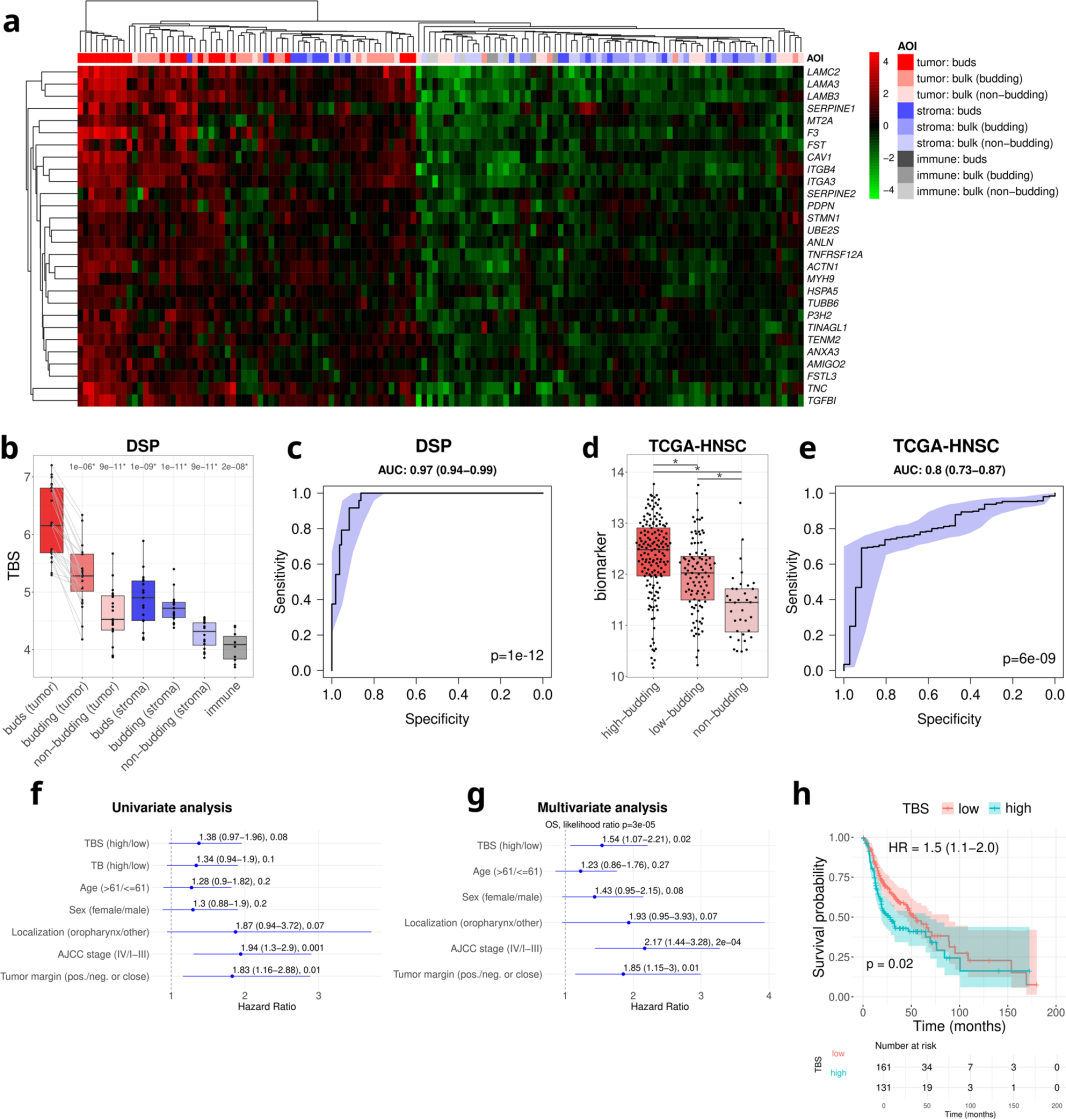
Tumor budding signature (TBS) in spatial transcriptomics and projection to bulk RNA-seq data from TCGA-HNSC. **a** Heatmap showing the expression patterns of the 28 genes composing the TBS. **b** TBS in the spatial transcriptomics compartments and **c** accompanying ROC separating tumor buds from all other compartments. **d** TBS of TCGA-HNSC samples stratified by their tumor budding (TB) activity (non-budding: *TB* = 0; budding: *TB* > 0, with low-budding: 0 < *TB* < 6 and high-budding: *TB* ≥ 6) and **e** accompanying ROC separating budding and non-budding TCGA-HNSC samples. **f** Univariate analysis of survival TBS (median split) and TB activity (median split) using the TCGA-HNSC data and **G** the corresponding multivariate survival analysis. **h** Kaplan-Meier curve of the TCGA-HNSC stratified by a threshold-optimized TBS

To develop a molecularly-defined tumor budding signature (TBS), we performed DGEA comparing tumor buds against all other defined segment types and compartments (as shown in detail in Additional File 1: Fig. S1c). The intersection of all sets of DEGs included 28 genes that were overexpressed in tumor buds relative to all other compartments. Based on this finding, TBS was defined as the mean expression of the 28 genes. The TBS genes were enriched for the apical junction (FC = 7.8, *p* = 3.5e-06), hypoxia (FC = 6.1, *p* = 7.9e-05), and epithelial-mesenchymal transition (EMT; FC = 5.1, *p* = 2.4e-05) hallmark pathways. Hierarchical clustering of the samples based on the TBS (Fig. 3a) in the spatial transcriptomics cohort showed that tumor buds tend to cluster separately from other segment types and compartments, with a TBS significantly higher than any other segment and compartment (Fig. 3b). ROC curve analysis confirmed TBS’s discriminatory power (AUC = 0.97, 95% CI: 0.94–0.99, *p* = 1e-12, Fig. 3c). Switching from the level of bud detection to the level of tumor classification, TBS effectively stratified budding versus non-budding tumors in the bulk RNA-seq TCGA-HNSC dataset (AUC = 0.8, 95% CI: 0.73–0.87, *p* = 6e-09, Fig. 3d-e), demonstrating its robustness across datasets and applicability in bulk RNA-seq data.

Next, we analyzed the prognostic value of the TBS in HPV-negative TCGA-HNSC using two cutoffs to define TBS-high and -low groups. Using the median as cutoff, we observed a non-significant trend for worse overall survival (OS) of TBS-high patients (HR = 1.38, 95% CI: 0.97–1.96, *p* = 0.08, Fig. 3f). In a subsequent multivariate analysis using the median as cutoff and adjusting for age, sex, tumor location, and AJCC stage, TBS was an independent prognostic factor for OS (HR = 1.54, *p* = 0.02, Fig. 3g). After TBS cutoff optimization using CutoffFinder for optimal patient stratification, the Kaplan-Meier survival analysis further confirmed that TBS-high patients showed significantly worse OS (HR = 1.5, 95% CI: 1.1-2.0, *p* = 0.02, Fig. 3h).

### TBS correlates with subtypes of EGFR activity

**Fig. 4:**
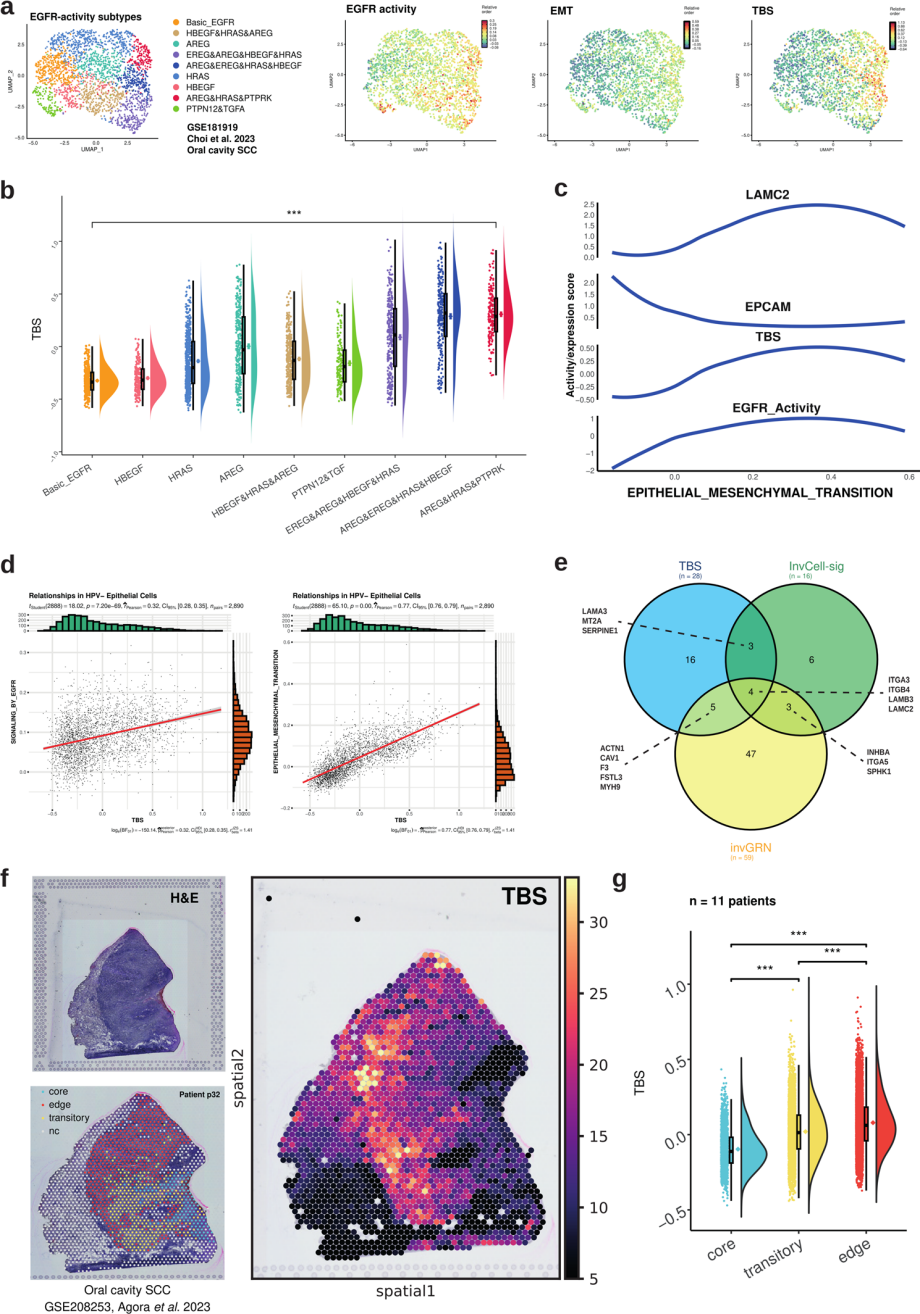
TBS correlates with EGFR-activity subtypes and is enhanced at the tumor leading edge. **a** EGFR-activity subtypes were inferred in HPV-neg. single malignant cells of GSE181919 by NMF using the REACTOME EGFR activity signature (*n* = 50 genes). Subtypes are depicted in a uMAP along with inferred EGFR activity, EMT score, and TBS. **b** The TBS (centered, SD-normalized) is depicted across HPV-neg. single malignant cells within the indicated EGFR-subtypes. *** indicates a *p*-value < 0.001 (one-way ANOVA). **c** EMT scores, EGFR-activity, TBS (centered, SD-normalized), and EpCAM and LAMC2 expression are depicted for all HPV-neg. malignant cells of GSE181919. **d** Correlation of the TBS (centered, SD-normalized) with EGFR signaling (left) and EMT (right) are shown as dot plots with Pearson correlation values. **e** Shown is a Venn representation of the intersection of genes from the TBS, the invasive cell signature (invCell-sig, and the invasive gene regulatory network (invGRN) (both described in Zhou et al. 2025 [26]). **f** Representative example of the TBS distribution within the tumor core, the leading edge, and the transitory regions of HNSCC. Shown are an H&E staining, a heatmap of the TBS, and a spot annotation for patient 32 of GSE208253. **g** The TBS (centered, SD-normalized) for tumor core, the leading edge, and the transitory regions of HNSCC (*n* = 11) of GSE208253 are depicted as raincloud plots

Receptor-mediated signaling pathways are crucial regulators of gradual forms of EMT and local invasion in HNSCC. We focused on EGFR as an approved therapeutic target for the treatment of recurrent or metastatic HNSCC (R/M-HNSCC) patients and based on the observed correlation with the TBS. A recently published [26] stratification of EGFR-activity in distinct subtypes identified at single-cell level in HNSCC was interrogated for correlations with the TBS. EGFR-activity subtypes were visualized in malignant cells of the scRNA-seq GSE181919 dataset, accounting for nine subtypes with differential expression of EGFR ligands. Highest EGFR activity was observed upon accumulated expression of amphiregulin (*AREG*) and epiregulin (*EREG*), which positively correlated with EMT and TBS at single-cell level (Fig. 4a-c, Additional File 1: Fig. S8). EMT scores positively correlated with EGFR activity, TBS, and *LAMC2* expression, while they negatively correlated with pan-carcinoma marker Epithelial Cell Adhesion Molecule (EpCAM) (Fig. 4c-d).

To address molecular overlaps between TBS and EGFR-mediated local invasion, we intersected the 28 genes of the TBS with an invasive cell-signature (invCell-sig) and with an invasive cell gene regulatory network (invGRN), both derived from transcriptomic analysis of locally invasive cells after EGF treatment [26]. Four genes were common to all signatures: *ITGA3* (integrin alpha 3), *ITGB4* (integrin beta 4), *LAMB3*, and *LAMC2* (Fig. 4e), the ITGB4/Laminin 5-axis being essential in EGFR-mediated local invasion [23, 26]. Thus, the TBS is strongly related to EGFR signaling, EMT, and induction of local invasion. Additionally, TBS displayed enhanced expression levels at the leading edge (LE) of HNSCC compared to transitory and tumor core (TC) areas (Fig. 4f-g, Additional File 1: Fig. S9).

### TBS expression marks malignant HNSCC cells

**Fig. 5:**
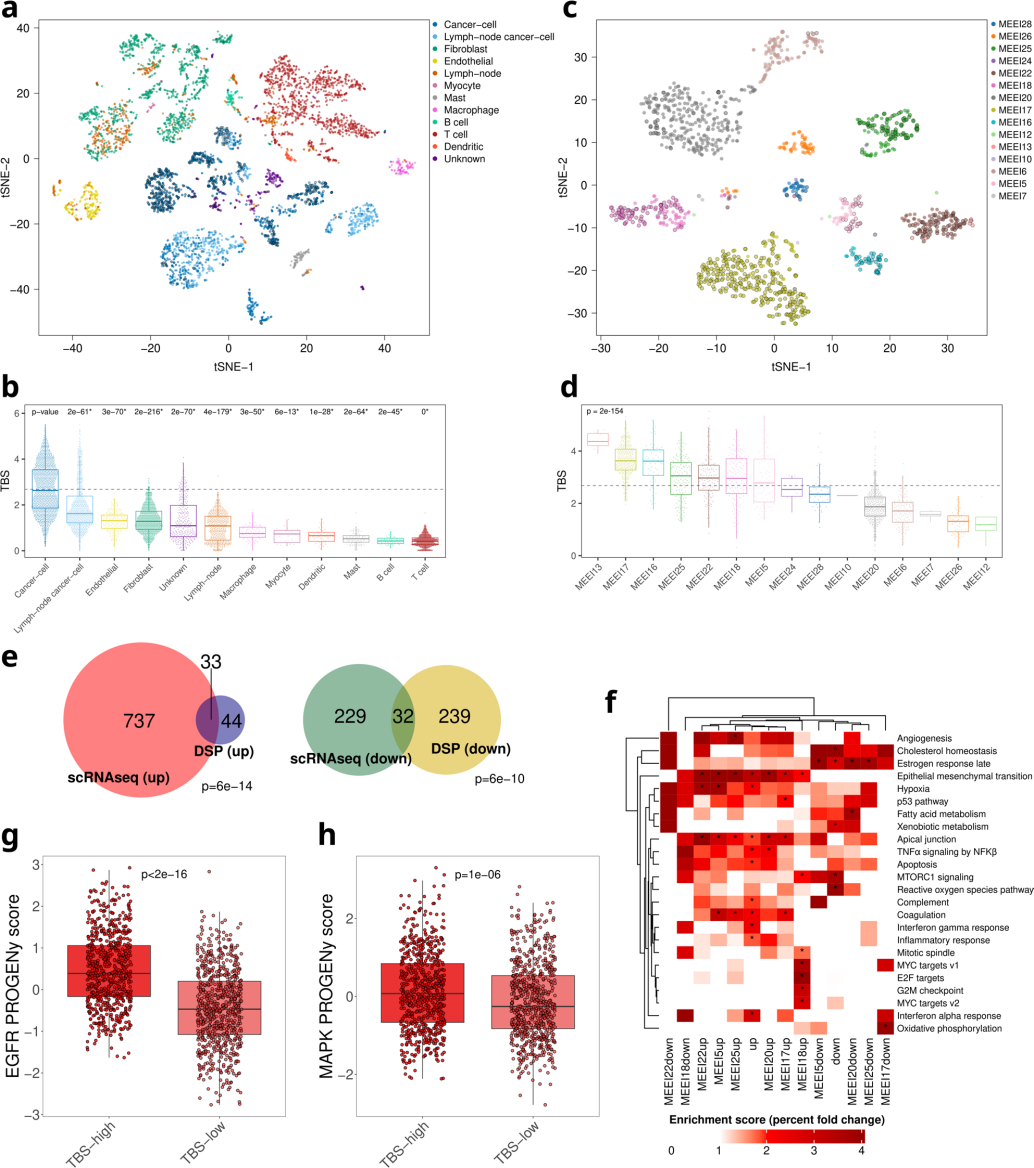
scRNA-seq data and tumor budding signature (TBS). **a** tSNE plot using all cell types and all patients. TBS-high cells are marked with a black circle around them. **b** TBS by cell type. The horizontal line shows the threshold used for cell classification in the TBS-low and TBS-high groups. **c** tSNE plot using all primary cancer cells from all patients. TBS-high cells are marked with a black circle around them. **d** TBS of primary cancer cells by patient. The horizontal line shows the threshold used for cell classification in the TBS-low and TBS-high groups. **e** Overlap between the spatial transcriptomics differentially expressed genes (DEGs) between the tumor buds and tumor bulk and the DEGs of the TBS-high and TBS-low primary cancer cells. Upregulated and downregulated DEGs are separately shown. **f** Overall (pooled patients) and per-patient enrichment analysis using the DEGs between TBS-high and TBS-low primary cancer cells. Only patients with at least 20 primary cells in the TBS-high and TBS-low groups were included in the per-patient analyses. **g** EGFR PROGENy pathway activation scores in the TBS-high and TBS-low groups. **h** MAPK PROGENy pathway activation scores in the TBS-high and TBS-low groups

To further investigate the TBS at the single-cell level, we analyzed the HNSCC scRNA-seq dataset from Puram et al. 2017 [13] computing the TBS for each cell. Highest scores were observed in cancer cells, particularly those from primary tumors, whereas stromal and immune cell populations exhibited significantly lower scores (Fig. 5a-b). The distribution of the TBS in primary cancer cells was bimodal, allowing a stratification of malignant cells into TBS-high and TBS-low groups with a cutpoint of TBS = 2.68 as the value where the two distributions cut each other (Fig. 5b). Some tumors contained a substantial proportion of TBS-high cells, while others exhibited a predominantly TBS-low phenotype, suggesting significant interpatient variability (Fig. 5c). The distribution of TBS within each tumor (Fig. 5d) further underscores this heterogeneity. These results were further validated with the HNSCC scRNA-seq dataset from Choi et al. 2023 [19]. Using cells from the HPV-negative, as well as the leukoplakia and normal samples, the highest TBS score was observed in the cancer cells, followed by the endothelial and epithelial cells, while it was generally low in the immune cells (Additional File 1: Fig. S10a) and varied by patient (Additional File 1: Fig. S10b). Additionally, TBS exhibited a progressive increase from epithelial cells of normal tissues (lowest TBS), to epithelial cells of leukoplakia tissues (intermediate TBS), to cancer cells (highest TBS, Additional File 1: Fig. S10c-d), although these results should be interpreted with caution due to the limited sample size, especially of the leukoplakia samples (*n* = 4).

Furthermore, we performed DGEA comparing the TBS-high and TBS-low primary tumor cancer cells, first across all patients and then stratified by patient. We identified 1271 DEGs between the TBS-high and TBS-low groups, excluding genes contained in the TBS, with a significant overlap of 65 DEGs (33 overexpressed and 32 underexpressed) with the spatial transcriptomics intratumoral DEGs (*p* = 6e-14 and *p* = 6e-10 respectively, Fig. 5e) and 110 DEGs (94 overexpressed and 16 underexpressed) with the Choi et al. scRNA-seq dataset (*p* < 2e-16 and *p* = 9.6e-16 respectively, Additional File 1: Fig. S10e-f). DEGs were significantly enriched for pathways involved in EMT, apical junctions, and coagulation when comparing the TBS-high and TBS-low primary tumor cells of all patients (pooled), as well as in the patient-specific DGEA (Fig. 5f), consistent with the spatial transcriptomics results. Furthermore, while no difference in the PI3K pathway activity scores was observed (*p* = 0.3), TBS-high cells showed increased EGFR [Hodges-Lehmann estimator = 0.88 (95% CI: 0.78–0.98), *p* < 2.e-16] and MAPK [Hodges-Lehmann estimator = 0.27 (95% CI: 0.16–0.38), *p =* 1e-06] activities relative to TBS-low cells (Fig. 5g-h). These results independently confirm the pronounced EGFR/MAPK signaling landscape observed in the tumor buds at single-cell level.

### Tumor budding is associated with a pEMT transcriptional program in Spatial transcriptomics and scRNA-seq data

**Fig. 6:**
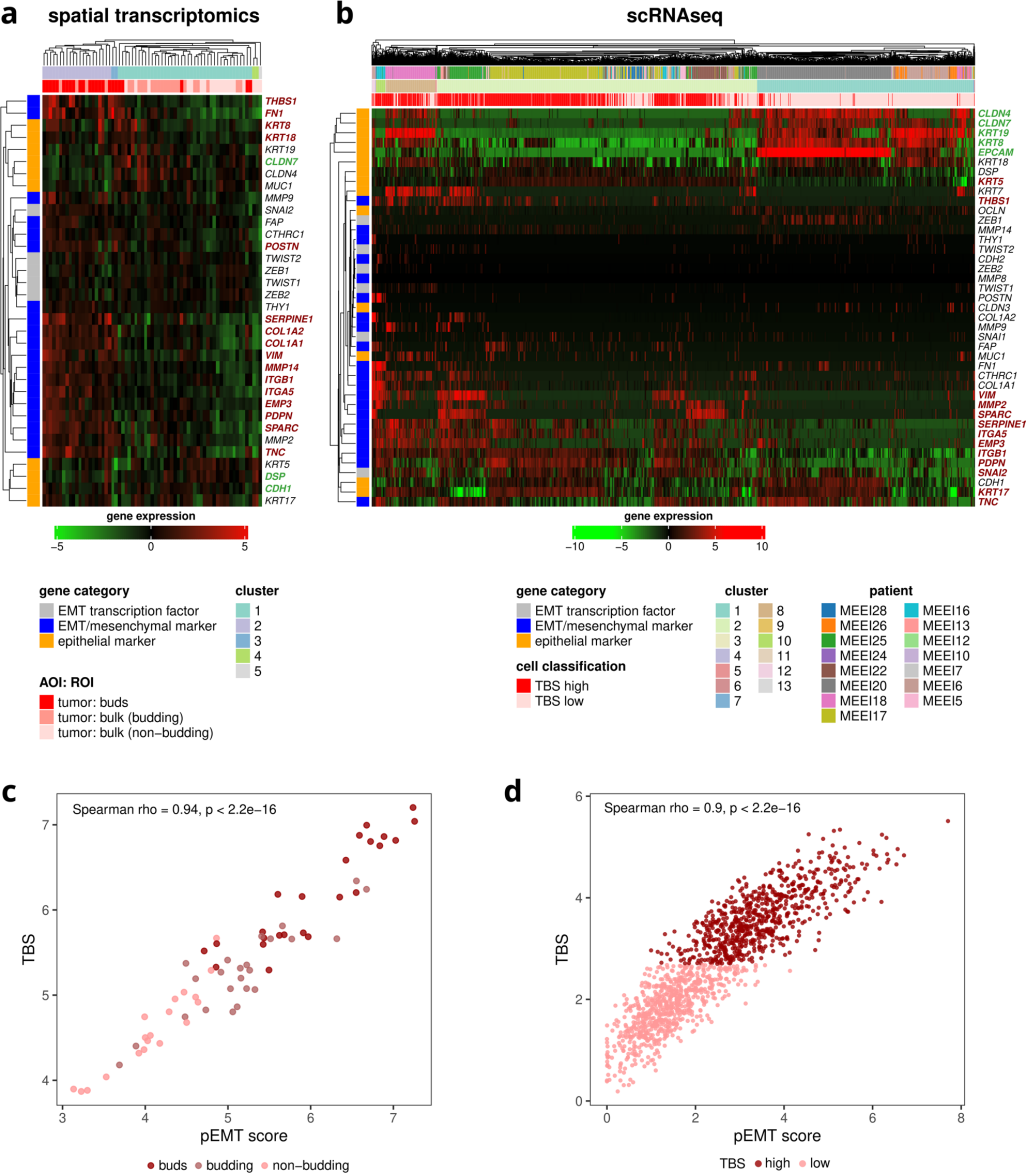
Evaluation of partial EMT (pEMT) phenotypes. **a** Epithelial and mesenchymal profiling of the spatial transcriptomics compartments and **b** the primary cancer cells from the scRNA-seq HNSCC dataset. Genes in red were overexpressed DEGs in tumor buds (spatial transcriptomics intratumoral comparison) or in TBS high cells (scRNAseq), while genes in green were underexpressed DEGs. **c** Correlation of TBS and the 15-gene pEMT score of tumor ROIs/AOIs in the spatial transcriptomics dataset. **d** Correlation of TBS and the 15-gene pEMT score of primary tumor cells in the scRNAseq dataset (GSE103322)

In the spatial transcriptomics dataset, tumor buds exhibited a partial EMT (pEMT) phenotype, characterized by the co-expression of epithelial and mesenchymal markers (Fig. 6a). Specifically, analyzing predefined curated sets of epithelial and mesenchymal genes, tumor buds showed increased expression of mesenchymal genes, including the collagen genes *COL1A1* and *COL1A2*, *MMP14*, Integrin alpha-5/beta-1 (*ITGA5* and *ITGB1*), *TNC*, Secreted Protein Acidic And Cysteine Rich (*SPARC*), Thrombospondin 1 (*THBS1*), *PDPN*, *SERPINE1*, and Vimentin (*VIM*), while retaining or even slightly increasing the expression of epithelial markers such as the keratins *KRT8* and *KRT18*. Other epithelial markers, such as Claudin 7 (*CLDN7*), *DSP*, and Cadherin 1 (*CDH1*) were overexpressed in the tumor buds. The mixture of epithelial and mesenchymal gene expression supports the pEMT state of the tumor buds.

To validate these findings at the single-cell level, we analyzed the expression of the same gene set in the scRNA-seq data derived from Puram et al. (Fig. 6b). Similarly to the spatial transcriptomics analysis, TBS-high primary tumor cancer cells exhibited overexpression of mesenchymal markers like *ITGA5* and *ITGB1*, *TNC*, *SPARC*, *THBS1*, *PDPN*, *SERPINE1*, and *VIM*, (but not *MMP14*, *COL1A2*, and *COL1A1*) along with retaining or increasing the expression of epithelial markers such as the keratins *KRT5* and *KRT17*, while other epithelial markers such as Claudin 4 (*CLDN4*), *CLDN7*, *KRT19*, and *EPCAM* were downregulated. These results from the scRNAseq data further support the presence of a pEMT state at the single-cell level. Importantly, Snail Family Transcriptional Repressor 2 (*SNAI2* or *SLUG*) was significantly overexpressed in TBS-high cells, suggesting that this EMT-TF plays an active role in driving pEMT.

To further quantify the association between tumor budding and pEMT, we applied a previously published 15-gene pEMT signature derived from Puram et al. to both spatial transcriptomics and scRNA-seq data (Fig. 6c-d). There is a notable overlap between the pEMT and TBS genes, with seven out of the 15 pEMT genes (*SERPINE1*, *TGFBI*, *LAMA3*, *LAMB3*, *LAMC2*, *PDPN*, *TNC*) being part of the TBS. Additionally, the pEMT score highly correlated with the TBS in both datasets (Spearman’s ρ = 0.94 and 0.90, respectively; *p* < 2.2 × 10⁻¹⁶), including in individual patients (Additional File 1: Fig. S11). These results further point towards the presence of a pEMT phenotype in tumor buds and TBS-high cancer cells using an established transcriptional program.

### TBS associates with drug response in squamous cell carcinoma cell lines

**Fig. 7:**
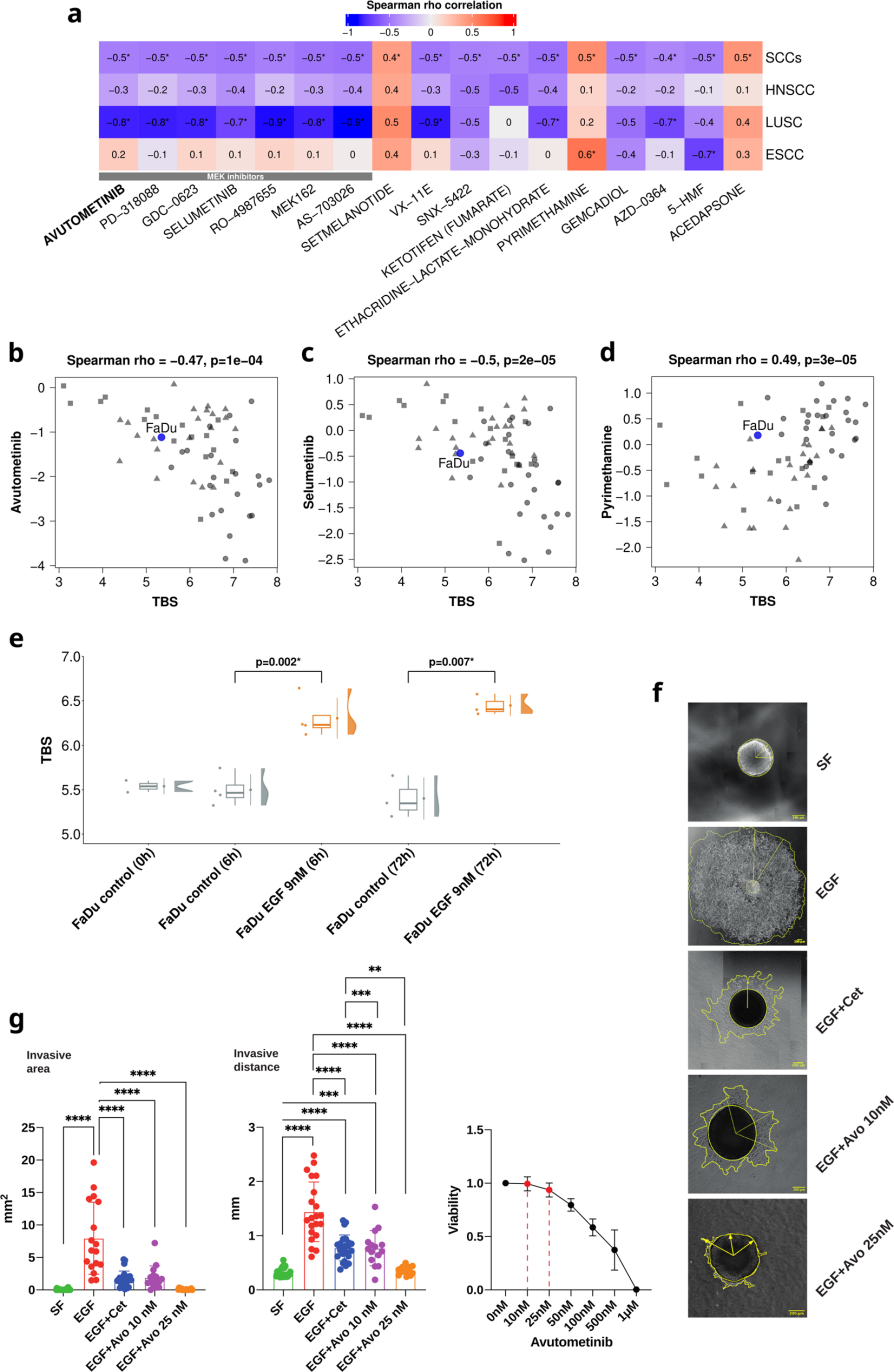
Drug responses of SCC cell lines by tumor budding signature (TBS). **a** Overview of the Spearman correlation coefficients for the pooled SCCs and for each cancer entity separately. MEK inhibitors are marked with a grey bar. Asterisks indicate significance after multiple testing correction (FDR 5%). **b-d** Selected examples of drugs for which the cell lines’ TBS correlated with their responses to the drug. The x axis refers to the log2-fold-change viability values from PRISM. The circles refer to HNSCC, the triangles to esophageal squamous cell carcinoma, and the squares to lung squamous cell carcinoma cell lines. The FaDu cell line is marked in blue. **e** TBS score of the FaDu cell line under serum-free (FaDu control) and EGF-treated (FaDu EGF 9nM) conditions after six and 72 h. **f** Local invasion induced by EGF treatment was assessed in a 3D-model of FaDu cell spheroid embedded in collagen I. Shown are representative micrographs of FaDu spheroids in the absence of growth factors (SF; serum-free) and after treatment with 9 nM EGF. Co-treatment with cetuximab and avutometinib are indicated. Yellow lines denote the original spheroid, invasive area, and invasive distance. The viability of FaDu cells after avutometinib treatment at increasing concentrations was assessed by WST-8 assay. Shown are mean with SD from *n* = 3 independent experiments. **g** Invasive area and distance of FaDu cell spheroid treated with the indicated compounds was quantified and is represented as mean with SD from *n* = 3 independent experiments performed with minimum five spheroids per treatment and experiment. SF: serum-free; EGF: EGF treatment only; EGF + Cet: EGF and cetuximab co-treatment; EGF + Avo 10 nM: EGF and avutometinib (10nM) co-treatment; EGF + Avo 25 nM: EGF and avutometinib (25nM) co-treatment. *P*-value ** < 0.01, *** < 0.001, **** < 0.0001

To investigate the potential of TBS as a predictive marker, we analyzed pharmacogenomic data from the Dependency Map portal. The analysis was performed in a pan-SCC panel including cell lines from head and neck, lung, and esophageal squamous cell carcinomas (SCCs). In the pan-SCC panel, high TBS was associated with greater sensitivity to MEK inhibitors, including avutometinib and selumetinib (Fig. 7a-c), while the opposite was observed for other compounds like pyrimethamine (Fig. 7d). Based on the findings of a correlation of TBS with EGFR-activity and an enhanced sensitivity of TBS-high cell lines to MEK inhibitors, we tested the impact of avutometinib in a functional 3D-assay of local invasion in HNSCC cells. Spheroids of FaDu cells were embedded in collagen I matrix and treated with EGF concentrations reportedly inducing EMT and local invasion [23, 26]. Under serum-free conditions, FaDu cells showed low TBS (Fig. 7e) and no spontaneous local invasion (Fig. 7f-g), whereas EGF-treatment promoted a marked increase in TBS (Fig. 7e) and strong ECM invasion (Fig. 7f-g). Co-treatment of spheroids with EGF and cetuximab confirmed the EGFR-specificity of the observed invasion (Fig. 7f-g). Importantly, co-treatment with RAF-MEK inhibitor avutometinib strongly (10 nM) or entirely (25 nM) blocked local invasion of FaDu cells without any measurable effect on vitality (Fig. 7f-g). Thus, both the analysis of the pan-SCC cell line panel and the invasion assays were in line with an activity of MEK inhibitors in TBS-high SCCs.

## Discussion

TB has emerged as a critical histopathological feature associated with poor prognosis across multiple cancer types, including HNSCC [4, 10]. In this study, we employed spatial transcriptomics and integrated them with bulk RNA-seq, scRNA-seq, and pharmacogenomic data to comprehensively characterize the transcriptional landscape of TB in HPV-negative HNSCC.

Spatial transcriptomics analysis revealed that TB is associated with a transcriptional program linked to invasive phenotypes and EGFR/MAPK pathway activity. The observed strong overexpression of *LAMA3*, *LAMB3*, and *LAMC2* in tumor buds further supports the role of laminin-332 in driving tumor invasion, consistent with previous studies demonstrating its presence in tumor budding areas and stroma-invading cancer cells, its enhancing of invadopodia formation and ECM degradation, and its correlation with aggressive oral squamous cell carcinoma (OSCC) phenotypes [62–64]. Functionally, blockade of the interaction of laminin-322 with its receptor ITGB4 as well as knock-down and knockout of the receptor strongly diminished local invasion of HNSCC cell lines in a 3D-model [23, 26]. TB was also associated with stromal alterations. The upregulation of *MMP14* and *INHBA* in the stroma of budding tumors aligns with prior reports of stromal remodeling facilitated by matrix metalloproteinases promoting ECM breakdown and cancer cell invasion [65, 66]. The downregulation of genes commonly linked to inflammatory signaling, immune cell recruitment, and tumor-promoting inflammation such as *S100A7*, *S100A8*, *S100A9* [67], and *CXCL14* [68, 69] in the stroma adjacent to tumor buds suggests a suppression of inflammatory and immune responses in regions immediately surrounding budding cells.

The regulatory network rewiring in buds highlights *JUP*, *CAV1* and *MMP14* as central hubs. Knockdown of *JUP* has been shown to promote EMT, cancer cell migration and invasion, and increased EGFR activation accompanied by increased AKT/GSK3β/β-catenin signaling activity in gastric cancer [70]. Our results support the above by showing that *JUP*’s association with epithelial and desmosomal gene downregulation links to the partial loss of the epithelial phenotype and to the destabilizing of cell-cell junctions in TB. *CAV1* expression has been reported as both tumor suppressing and promoting, depending on the context [71]. The spatial transcriptomics results show that in the context of HNSCC, *CAV1* is a central hub and upregulated in tumor buds and, by extension, linked to increased tumor invasion potential, an observation supported by a recently published study on independent cohorts, including a spatially-resolved IHC-based analysis showing increased CAV1 protein expression in the tumor buds in HNSCC [12]. *MMP14*’s expanded connectivity to *TGFBI* and *TNC* in tumor buds mirrors its established function as a matrix metalloproteinase in ECM remodeling [72].

The differential correlation of gene expression analysis, having identified hundreds of differentially correlating gene pairs as well as disrupted and novel hub genes, supports the notion that TB is driven by a complex rewiring of regulatory relationships. The observed loss of strong correlations between certain tumor-tumor gene pairs in buds, such as *RPS2*-*MMD*, may reflect a decoupling of translational control. Ribosomal Protein S2 (*RPS2*) encodes a ribosomal protein involved in translation regulation, while *MMD*, despite being known as a macrophage-derived marker, has been reported as overexpressed in cancer cells compared to normal tissue, facilitating increased tumor cell proliferation [73] and interacting with RAS leading to sustained ERK signaling [74]. Conversely, the emergence of new correlations, like the *PDE7A*-*GLIPR2* pair in tumor buds, highlights the activation of gene modules associated with migration, invasion, and EMT [75–77]. Network remodeling was observed in the stromal compartment as well, suggestive of stromal cells actively adapting in response to or facilitating budding. Additionally, the observed alterations of tumor-stroma gene correlations reinforce the concept of active crosstalk between compartments. Such an example is the emergence of a new correlation between stromal *TRPM3* and tumoral *SQSTM1* expression in tumor buds, underscoring the interplay of TME relationships in TB connected to ECM remodeling, EMT, and MAPK signaling alterations [78–80]. The Transient Receptor Potential Cation Channel Subfamily M (TRPM) family of proteins has been implicated in the transmission of signals from the TME, tumor progression, ECM remodeling, EMT, and signaling pathways, including MAPK [80–82] and Sequestosome 1 (*SQSTM1*) is implicated in tumorigenesis and can regulate the activation of NFKB1 [78, 79].

Extending beyond earlier bulk and scRNA-seq studies [12, 13], the spatial transcriptomics enabled to reveal a biomarker of tumor buds, the 28-gene TBS. The TBS was highly effective at distinguishing tumor buds from any other segment type and compartment in spatial transcriptomics data and at separating budding from non-budding tumors in bulk RNA-seq data. TBS demonstrated independent prognostic significance in multivariate survival analysis, with high TBS correlating with poorer overall survival. Numerous studies have shown that TB correlates with worse prognosis in HNSCC [4, 10]. Our results extend these findings providing a molecularly defined biomarker for TB that can serve as a quantitative measure for risk stratification in HNSCC.

Analysis of two scRNA-seq datasets and independent spatial transcriptomics data confirmed that the TBS is predominantly expressed in malignant epithelial cells at the leading edge of tumors. The observed bimodal distribution of TBS in primary cancer cells is in line with the superposition of budding and non-budding cell populations. This heterogeneity aligns with prior reports indicating that EMT programs are heterogeneously activated within tumors [83].

Our data comprehensively characterized the pEMT state of tumor buds. Tumor buds upregulate mesenchymal markers and downregulate *CDH1* while retaining epithelial markers. Additionally, TBS showed strong correlation with a validated 15-gene pEMT signature from Puram et al. [13], further substantiating a pEMT phenotype of tumor buds. scRNA-seq further confirmed these findings, showing that TBS-high cells express largely the same mesenchymal genes while selectively retaining epithelial traits. The above are in line with prior studies linking tumor budding to pEMT across several cancer types, including HNSCC, colorectal, pancreatic, and esophageal cancers [84]. In HNSCC, tumor buds have been shown to coexpress mesenchymal markers such as *VIM* and downregulate *CDH1* concurrently preserving other epithelial features and pEMT profiles were associated with lymph node metastasis, much like TB [85, 86]. Our results extend these observations by providing detailed spatial and single-cell characterization of the pEMT state of tumor buds in HNSCC. Additionally, we provide evidence for a link between newly described EGFR-activity subtypes that induce pEMT and local invasion [26] with the TBS. AREG- and EREG-dependent EGFR signaling associates with enhanced expression of the TBS, and, reciprocally, the TBS comprised several genes described as instrumental modulators of EGFR-dependent local invasion such as ITGB4 and its ligand laminin-332. Importantly, high expression of *ITGB4* and *LAMC2* in R/M-HNSCC patients predicted an improved progression-free survival after cetuximab treatment [23, 26]. It is therefore tempting to speculate that a blockade of EGFR may impact TB and, thereby, the clinical performance of (R/M)-HNSCC patients.

Analyzing drug sensitivity data of a large panel of SCC cell lines, the effectiveness of MEK inhibitors stood by interacting with the level of the TBS, in line with the observation that MEK inhibition suppresses EMT and invasion in Laminin γ2 overexpressing HNSCC cells [87]. Additionally, a recent study identified a gene regulatory network driving EGFR-mediated invasion in HNSCC that includes key EMT effectors, many of which are utilized inferring the TBS, and is suppressed by MEK inhibition [26]. Furthermore, in a recent phase 1 FRAME trial, MAPK/MEK inhibition with avutometinib together with defactinib has shown favorable clinical outcomes in RAS/RAF-altered non-small cell lung cancer and low-grade serous ovarian cancer [88] and in 3D cell culture models, blocking MEK activity reduced invasive behavior, supporting MEK-ERK signaling as a therapeutic vulnerability in pEMT-like, locally invasive tumor phenotypes [26]. Consistent with the cited studies, the RAF-MEK inhibitor avutometinib exhibited outstanding blocking capacity in the studied 3D-model mimicking initial phases of EGFR-mediated local invasion in HNSCC.

Lastly, while benefiting from the cutting-edge technology of DSP with the WTA for spatial transcriptomics, there are certain limitations. DSP lacks single-cell resolution, limiting the spatial resolution of the analysis and potentially obscuring heterogenous gene expression within the investigated regions. The WTA panel is confined to a predefined gene set, limiting detection of unrepresented transcripts and incurring probe dropouts. Additionally, the NanoString GeoMx DSP's ROI size constraint (660 μm × 785 μm) made finding samples with sufficient tumor bud density challenging. Samples meeting density requirements exhibited high tumor budding scores, potentially biasing the analyses towards highly budding cases. Despite above, integrating the DSP results with analyses of bulk RNA-seq, scRNA-seq, and independent spatial transcriptomics datasets provided independent cross-platform and cross-cohort validations, strengthening the validity and robustness of the study.

## Conclusions

This study includes the first well-powered spatial transcriptomics analysis of TB in HNSCC at the genomic scale, revealing a novel 28-gene tumor budding signature (TBS) that distinctively characterizes tumor buds. The TBS correlated strongly with gene expression signatures of pEMT, separated budding and non-budding tumors, and was associated with unfavorable patient outcome in HPV-negative HNSCC. In cell culture models, the level of the TBS was a predictive marker for MEK inhibition including the RAF-MEK inhibitor avutometinib. The study delivered new insights in the molecular underpinnings of TB in HNSCC and its role for risk stratification and therapy guidance. With a growing role of immune checkpoint inhibition in HNSCC (e.g., KEYNOTE-689 [89], NIVOPOSTOP [90] trials), TBS’s predictive role for immunotherapy warrants further investigation.

## Supplementary Information

Additional file 1. Tables S1-S2 and figures S1-S11. Table S1. Clinicopathological characteristics of the in-house spatial transcriptomics cohort. Table S2. TCGA-HNSC cases with corresponding tumor budding values. Fig. S1. Quality control and normalization metrics of spatial transcriptomics data. Fig. S2. Expression levels of immune (PTPRC/CD45) and epithelial keratin transcripts across tumor, stromal, and immune compartments. Fig. S3. Heatmap and clustering of samples based on PROGENy pathway activity scores. Fig. S4. Stromal differential gene expression analyses. Fig. S5. Gene regulatory networks in tumor buds and in tumor bulk using ARACNe-inferred TCGA-HNSC regulatory interactions. Fig. S6. Differential gene correlation networks between tumor buds and tumor bulk (networks with 2 to 8 members). Fig. S7. Differential gene correlation networks between tumor buds and tumor bulk (networks with 2 members). Fig. S8. Expression of tumor budding signature genes across EGFR activity subtypes in single malignant cells (scRNA-seq dataset GSE181919). Fig. S9. Spatial distribution of tumor budding signature scores across tumor regions in spatial transcriptomics dataset GSE208253. Fig. S10. Association of tumor budding signature scores with cell types, patient samples, and along the HNSCC progression axis in the scRNA-seq dataset GSE181919. Fig. S11. Correlation between tumor budding signature scores and partial EMT scores in primary tumor cells across patients. 

## Data Availability

The in-house spatial transcriptomics dataset generated for this study, comprising data from 43 immunohistochemically p16-negative HNSCC patients, have been uploaded to the Gene Expression Omnibus (GEO) repository with accession number GSE300414 [17]. The TCGA-HNSC data are accessible via the Genomic Data Commons portal (https://portal.gdc.cancer.gov) and the TCGA PanCanAtlas publications [18]. The additional single-cell RNA sequencing and spatial transcriptomics datasets used in this study can be accessed via the GEO repository (Puram et al. 2017: GSE103322 [13], Choi et al. 2023: GSE181919 [19], Arora et al. 2023: GSE208253 [20]). FaDu cell line transcriptomics data before and after EGF treatment at six and 72 h were retrieved from the GEO repository GSE200421 [23]. The pharmacogenomic data can be downloaded from the Dependency Map (DepMap) portal [21]. The packages and tools utilized for data analysis and graphics generation are cited in the Methods section, along with a description of the options used. The code generated is available from the corresponding author upon request.

## Abbreviations

AOI: Area of illumination
AUC: Area under the curve
CD45: Cluster of Differentiation 45
CLDN: Claudin (e.g., CLDN3, CLDN4, CLDN7)
COL1A1/COL1A2: Collagen type I alpha 1/2 chain
DCC: Digital count counts (GeoMx output)
DEG: Differentially expressed gene
DGEA: Differential gene expression analysis
DSP: Digital spatial profiling
EGFR: Epidermal growth factor receptor
EMT: Epithelial-mesenchymal transition
FDR: False discovery rate
FFPE: Formalin-fixed paraffin-embedded
FN1: Fibronectin 1
GEO: Gene Expression Omnibus
H&E: Hematoxylin and eosin
HNSCC: Head and neck squamous cell carcinoma
HPF: High-power field
HPV: Human papillomavirus
ITGA5/ITGB1: Integrin subunit alpha 5/beta 1
KRT: Keratin (e.g., KRT5, KRT7, KRT8, KRT17, KRT18, KRT19)
LE: Leading edge
LOQ: Limit of quantification
MEK: Mitogen-activated protein kinase kinase
MSigDB: Molecular Signatures Database
NGS: Next-generation sequencing
NMF: Non-negative matrix factorization
NTC: Non-template control
OD: Optical density
pEMT: Partial epithelial-mesenchymal transition
PROGENy: Pathway RespOnsive GENes (pathway activity inference method)
Q3/UQ: Upper quartile normalization
ROI: Region of interest
ROC: Receiver operating characteristic
SCC: Squamous cell carcinoma
scRNA-seq: Single-cell RNA sequencing
SPARC: Secreted protein acidic and rich in cysteine
TB: Tumor budding
TBS: Tumor budding signature
TC: Tumor core
TCGA: The Cancer Genome Atlas
TCGA-HNSC: The Cancer Genome Atlas - Head and Neck Squamous Cell Carcinoma cohort
TNC: Tenascin C
tSNE: t-distributed stochastic neighbor embedding
uMAP: Uniform manifold approximation and projection
UMI: Unique molecular identifier
WTA: Whole transcriptome atlas
