# Supplementary material for: Spatial transcriptomics reveals a molecular tumor budding signature in head and neck cancer
Source: Genome Med. 2026 Apr 15;18:43. doi: 10.1186/s13073-026-01612-2 (PMC13081553; doi:10.1186/s13073-026-01612-2)
Supplement: Supplementary file 1 — Additional file 1. Tables S1-S2 and figures S1-S11. Table S1. Clinicopathological characteristics of the in-house spatial transcriptomics cohort. Table S2. TCGA-HNSC cases with corresponding tumor budding values. Fig. S1. Quality control and normalization metrics of spatial transcriptomics data. Fig. S2. Expression levels of immune (PTPRC/CD45) and epithelial keratin transcripts across tumor, stromal, and immune compartments. Fig. S3. Heatmap and clustering of samples based on PROGENy pathway activity scores. Fig. S4. Stromal differential gene expression analyses. Fig. S5. Gene regulatory networks in tumor buds and in tumor bulk using ARACNe-inferred TCGA-HNSC regulatory interactions. Fig. S6. Differential gene correlation networks between tumor buds and tumor bulk (networks with 2 to 8 members). Fig. S7. Differential gene correlation networks between tumor buds and tumor bulk (networks with 2 members). Fig. S8. Expression of tumor budding signature genes across EGFR activity subtypes in single malignant cells (scRNA-seq dataset GSE181919). Fig. S9. Spatial distribution of tumor budding signature scores across tumor regions in spatial transcriptomics dataset GSE208253. Fig. S10. Association of tumor budding signature scores with cell types, patient samples, and along the HNSCC progression axis in the scRNA-seq dataset GSE181919. Fig. S11. Correlation between tumor budding signature scores and partial EMT scores in primary tumor cells across patients. [file 13073_2026_1612_MOESM1_ESM.pdf]

Additional file 1 for

Spatial Transcriptomics Reveals a Molecular Tumor Budding Signature in Head and Neck Cancer

This file includes the following supplementary information:

Table S1,

Table S2,

Fig. S1,

Fig. S2,

Fig. S3,

Fig. S4,

Fig. S5,

Fig. S6,

Fig. S7,

Fig. S8,

Fig. S9,

Fig. S10

Fig. S11

Table S1. Characteristics of the in-house spatial transcriptomics cohort.

Case	Sex	Age	Localisation	pT	pN	Grading	TB 10 HPF	UICC stage
1_budding	F	73	Tongue	3	2a	3	7	IVA
1_nonbudding	F	74	Tongue	3	2b	2	0	IVA
2_budding	F	69	Floor of mouth	4a	X	3	6	IVA
2_nonbudding	F	74	Tongue	1	X	1	0	IVA
3_budding	M	61	Floor of mouth	2	2b	3	26	IVA
3_nonbudding	M	55	Tongue	1	0	1	0	I
4_budding	M	96	Upper jaw	4a	X	3	15	IVA
4_nonbudding	F	77	Lower jaw	2	0	1	0	II
5_budding	F	77	Floor of mouth	3	0	3	9	III
5_nonbudding	F	72	Floor of mouth	3	1	2	0	III
6_budding	M	57	Floor of mouth	3	0	2	14	III
6_nonbudding	M	59	Floor of mouth	4a	0	2	0	IVA
7_budding	M	58	Lip (oral)	3	3b	3	18	IVB
7_nonbudding	M	61	Lower jaw	4a	0	2	0	IVA
9_budding	M	63	Retromolar	2	0	2	8	II
9_nonbudding	M	53	Lower jaw	4a	0	2	0	IVA
11_budding	F	76	Lower jaw	2	3b	2	6	IVB
11_nonbudding	F	73	Upper jaw	4a	0	2	0	IVA
12_budding	M	88	Tongue	3	0	2	7	III
12_nonbudding	M	73	Lower jaw	pT4a	0	2	0	IVA
15_budding	M	67	Tongue	2	2b	3	6	IVA
15_nonbudding	M	51	Floor of mouth	4a	0	2	0	IVA
16_budding	M	53	Floor of mouth	2	0	2	6	II
17_budding	F	66	Floor of mouth	2	2b	2	14	IVA
19_budding	M	59	Lower jaw	pT4a	0	3	10	IVA
19_nonbudding	F	91	Floor of mouth	3	0	1	0	III
20_budding	M	56	Tongue	2	X	3	7	II
21_budding	M	60	Floor of mouth	pT4a	3b	3	17	IVB
23_budding	F	66	Tongue	3	0	3	6	III
23_nonbudding	M	69	Lower jaw	4a	2b	2	0	IVA
24_budding	M	78	Tongue	3	3b	3	11	IVB
25_budding	F	70	Floor of mouth	pT4a	1	2	10	IVA
25_nonbudding	M	55	Lower jaw	pT4a	1	2	0	IVA
27_budding	F	53	Tongue	3	0	3	8	III
27_nonbudding	F	71	Lower jaw	3	X	2	0	III
30_budding	M	75	Tongue	2	X	2	9	II
30_nonbudding	M	72	Cheek (oral)	2	X	1	0	II
31_budding	M	50	Tongue	2	X	3	9	II
31_nonbudding	F	80	Upper jaw	2	X	1	0	II
32_budding	F	54	Lower jaw	pT4a	2a	3	7	IVA
32_nonbudding	M	49	Tongue	2	0	2	0	II
33_budding	M	53	Tongue	2	1	2	11	III
33_nonbudding	M	63	Upper jaw	2	1	2	0	III

Table S2. TCGA-HNSC samples with corresponding tumor budding (TB) values.

sample	TB	sample	TB	sample	TB	sample	TB
TCGA-4P-AA8J	17	TCGA-CN-6998	23	TCGA-CV-7252	6	TCGA-F7-A620	16
TCGA-BA-4074	24	TCGA-CN-A63T	22	TCGA-CV-7253	2	TCGA-F7-A622	3
TCGA-BA-4078	2	TCGA-CN-A63U	22	TCGA-CV-7254	16	TCGA-F7-A623	0
TCGA-BA-5555	0	TCGA-CN-A63V	0	TCGA-CV-7255	17	TCGA-H7-A6C4	7
TCGA-BA-5558	0	TCGA-CN-A642	1	TCGA-CV-7261	24	TCGA-HD-7229	5
TCGA-BA-6869	0	TCGA-CQ-5326	32	TCGA-CV-7263	3	TCGA-HD-7753	5
TCGA-BA-6873	18	TCGA-CQ-5331	0	TCGA-CV-7409	1	TCGA-HD-7831	14
TCGA-BA-7269	15	TCGA-CQ-6218	22	TCGA-CV-7411	3	TCGA-HD-7917	6
TCGA-BA-A6D8	3	TCGA-CQ-6219	4	TCGA-CV-7413	4	TCGA-HD-8224	0
TCGA-BA-A6DA	21	TCGA-CQ-6220	4	TCGA-CV-7414	2	TCGA-HD-8634	7
TCGA-BA-A6DB	17	TCGA-CQ-6222	4	TCGA-CV-7415	12	TCGA-HD-8635	29
TCGA-BA-A6DD	22	TCGA-CQ-6223	2	TCGA-CV-7418	3	TCGA-HD-A4C1	0
TCGA-BA-A6DE	4	TCGA-CQ-6224	20	TCGA-CV-7421	6	TCGA-HD-A6HZ	2
TCGA-BA-A6DG	23	TCGA-CQ-6227	9	TCGA-CV-7422	0	TCGA-HD-A6I0	2
TCGA-BA-A6DI	5	TCGA-CQ-6228	20	TCGA-CV-7423	3	TCGA-IQ-7631	0
TCGA-BA-A6DJ	8	TCGA-CQ-6229	13	TCGA-CV-7428	4	TCGA-IQ-7632	0
TCGA-BA-A8YP	10	TCGA-CQ-7067	17	TCGA-CV-7430	3	TCGA-IQ-A61E	9
TCGA-BB-4217	7	TCGA-CQ-A4C6	25	TCGA-CV-7432	3	TCGA-IQ-A61G	1
TCGA-BB-7862	8	TCGA-CQ-A4CH	3	TCGA-CV-7433	1	TCGA-IQ-A61H	6
TCGA-BB-7863	3	TCGA-CV-5434	10	TCGA-CV-7434	25	TCGA-IQ-A61J	22
TCGA-BB-7870	0	TCGA-CV-5435	9	TCGA-CV-7435	8	TCGA-IQ-A6SG	8
TCGA-BB-7872	15	TCGA-CV-5436	18	TCGA-CV-7438	28	TCGA-IQ-A6SH	1
TCGA-BB-8596	20	TCGA-CV-5439	3	TCGA-CV-A45Q	10	TCGA-KU-A66S	12
TCGA-BB-8601	2	TCGA-CV-5440	2	TCGA-CV-A45R	7	TCGA-KU-A66T	9
TCGA-BB-A5HU	27	TCGA-CV-5441	23	TCGA-CV-A45U	7	TCGA-KU-A6H8	7
TCGA-BB-A5HY	16	TCGA-CV-5444	9	TCGA-CV-A45W	1	TCGA-P3-A5Q6	2
TCGA-BB-A5HZ	30	TCGA-CV-5970	1	TCGA-CV-A45X	0	TCGA-P3-A5QA	8
TCGA-BB-A6UO	10	TCGA-CV-5973	3	TCGA-CV-A45Y	16	TCGA-P3-A6SX	16
TCGA-C9-A47Z	8	TCGA-CV-5976	5	TCGA-CV-A45Z	9	TCGA-P3-A6T0	9
TCGA-CN-4722	10	TCGA-CV-5977	8	TCGA-CV-A460	8	TCGA-P3-A6T2	18
TCGA-CN-4723	16	TCGA-CV-5978	6	TCGA-CV-A461	0	TCGA-P3-A6T3	10
TCGA-CN-4725	2	TCGA-CV-6003	0	TCGA-CV-A463	4	TCGA-P3-A6T4	11
TCGA-CN-4727	5	TCGA-CV-6436	3	TCGA-CV-A464	1	TCGA-P3-A6T5	16
TCGA-CN-4728	5	TCGA-CV-6441	2	TCGA-CV-A465	4	TCGA-P3-A6T7	10
TCGA-CN-4729	3	TCGA-CV-6936	2	TCGA-CV-A468	6	TCGA-P3-A6T8	44
TCGA-CN-4731	1	TCGA-CV-6937	4	TCGA-CV-A6JD	4	TCGA-QK-A64Z	16
TCGA-CN-4733	80	TCGA-CV-6940	7	TCGA-CV-A6JM	11	TCGA-QK-A652	9
TCGA-CN-4734	1	TCGA-CV-6941	0	TCGA-CV-A6JN	9	TCGA-QK-A6IG	16
TCGA-CN-4736	7	TCGA-CV-6942	5	TCGA-CV-A6JO	9	TCGA-QK-A6IH	1
TCGA-CN-4737	4	TCGA-CV-6943	4	TCGA-CV-A6JT	13	TCGA-QK-A6II	18
TCGA-CN-4738	3	TCGA-CV-6948	0	TCGA-CV-A6JU	3	TCGA-QK-A6IJ	15
TCGA-CN-4739	0	TCGA-CV-6950	10	TCGA-CV-A6JY	0	TCGA-QK-A6VB	0
TCGA-CN-4740	0	TCGA-CV-6951	10	TCGA-CV-A6JZ	8	TCGA-QK-A6VC	17
TCGA-CN-4742	9	TCGA-CV-6952	13	TCGA-CV-A6K0	6	TCGA-QK-A8Z7	8
TCGA-CN-5355	4	TCGA-CV-6954	16	TCGA-CV-A6K1	4	TCGA-QK-A8Z8	0
TCGA-CN-5356	2	TCGA-CV-6955	11	TCGA-CV-A6K2	2	TCGA-QK-A8Z9	18
TCGA-CN-5358	8	TCGA-CV-6956	19	TCGA-CX-A4AQ	0	TCGA-QK-A8ZB	9
TCGA-CN-5359	3	TCGA-CV-6959	10	TCGA-D6-6516	5	TCGA-QK-AA3J	0

TCGA-CN-5360	0	TCGA-CV-6960	23	TCGA-D6-6517	39	TCGA-QK-AA3K	5
TCGA-CN-5361	8	TCGA-CV-6962	6	TCGA-D6-6823	9	TCGA-RS-A6TO	34
TCGA-CN-5363	19	TCGA-CV-7089	16	TCGA-D6-6824	5	TCGA-T2-A6WX	0
TCGA-CN-5364	4	TCGA-CV-7090	3	TCGA-D6-6825	8	TCGA-T2-A6WZ	14
TCGA-CN-5365	15	TCGA-CV-7091	11	TCGA-D6-6826	45	TCGA-T2-A6X2	3
TCGA-CN-5366	24	TCGA-CV-7095	20	TCGA-D6-6827	1	TCGA-TN-A7HJ	9
TCGA-CN-5367	17	TCGA-CV-7097	19	TCGA-D6-8568	9	TCGA-UF-A718	17
TCGA-CN-5373	0	TCGA-CV-7099	4	TCGA-D6-8569	4	TCGA-UF-A719	1
TCGA-CN-6010	5	TCGA-CV-7101	0	TCGA-D6-A4Z9	7	TCGA-UF-A71A	11
TCGA-CN-6011	5	TCGA-CV-7102	22	TCGA-D6-A4ZB	2	TCGA-UF-A71B	3
TCGA-CN-6013	3	TCGA-CV-7103	10	TCGA-D6-A6EO	0	TCGA-UF-A71D	0
TCGA-CN-6016	3	TCGA-CV-7104	20	TCGA-D6-A6EQ	11	TCGA-UF-A71E	5
TCGA-CN-6017	18	TCGA-CV-7177	12	TCGA-D6-A74Q	3	TCGA-UF-A7J9	13
TCGA-CN-6018	4	TCGA-CV-7178	4	TCGA-DQ-5625	9	TCGA-UF-A7JA	1
TCGA-CN-6019	2	TCGA-CV-7180	6	TCGA-DQ-7589	5	TCGA-UF-A7JC	5
TCGA-CN-6020	0	TCGA-CV-7183	6	TCGA-DQ-7595	0	TCGA-UF-A7JD	22
TCGA-CN-6021	6	TCGA-CV-7235	0	TCGA-F7-7848	2	TCGA-UF-A7JF	2
TCGA-CN-6023	5	TCGA-CV-7236	20	TCGA-F7-8298	16	TCGA-UF-A7JH	19
TCGA-CN-6024	17	TCGA-CV-7238	7	TCGA-F7-8489	24	TCGA-UF-A7JJ	7
TCGA-CN-6988	7	TCGA-CV-7242	3	TCGA-F7-A50G	0	TCGA-UF-A7JK	7
TCGA-CN-6989	4	TCGA-CV-7243	14	TCGA-F7-A50I	15	TCGA-UF-A7JO	4
TCGA-CN-6992	4	TCGA-CV-7245	10	TCGA-F7-A50J	5	TCGA-UF-A7JS	20
TCGA-CN-6994	3	TCGA-CV-7247	5	TCGA-F7-A61S	5	TCGA-UF-A7JT	6
TCGA-CN-6996	10	TCGA-CV-7248	12	TCGA-F7-A61V	2	TCGA-WA-A7GZ	17
TCGA-CN-6997	0	TCGA-CV-7250	0	TCGA-F7-A61W	12	TCGA-WA-A7H4	16

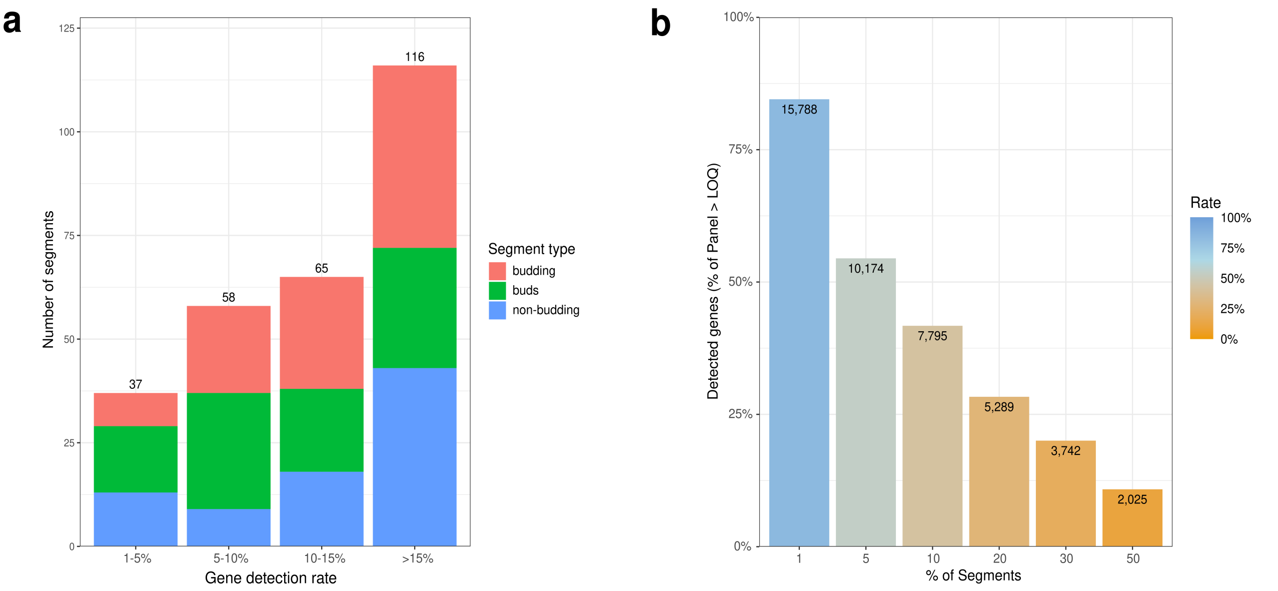

c

Class	Immune AOIs (Cases)	Stroma AOIs (Cases)	Tumor AOIs (Cases)	Dataset
**buds**	4 (2)	49 (24)	49 (24)	Initial
**buds**	0 (0)	34 (19)	43 (24)	Post-QC
**budding**	9 (5)	48 (24)	48 (24)	Initial
**budding**	6 (4)	39 (21)	47 (24)	Post-QC
**non-budding**	14 (9)	36 (19)	36 (19)	Initial
**non-budding**	8 (6)	29 (16)	33 (19)	Post-QC

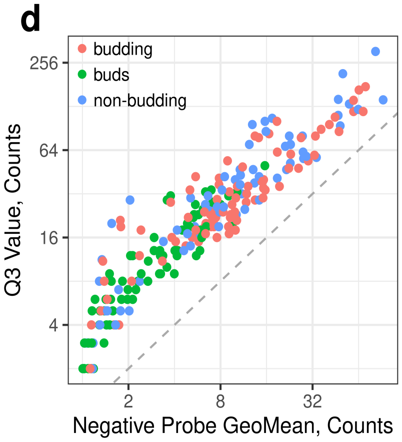

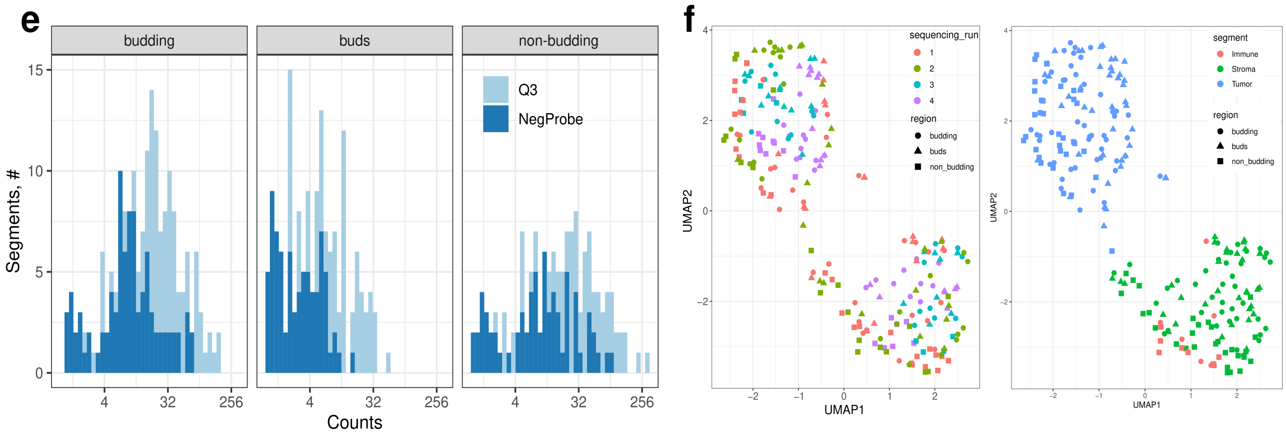

Fig. S1. Overview of the detected probes and ROIs/AOIs. a Number of segments by gene detection rate. **b** Genes detected above the limit of quantification (LOQ). **c** Pre- and post-QC number of segments per ROI/AOI category and class. **d** Q3 and negative probe counts per ROI/AOI after quality check (QC) filtering of probes and segments. **e** Distribution of upper-quartile normalized gene counts (Q3) and the corresponding distribution of the negative probes per ROI category. **f** UMAP of the samples after QC and normalization annotated for the ROI type, the sequencing run (left) and compartment (right).

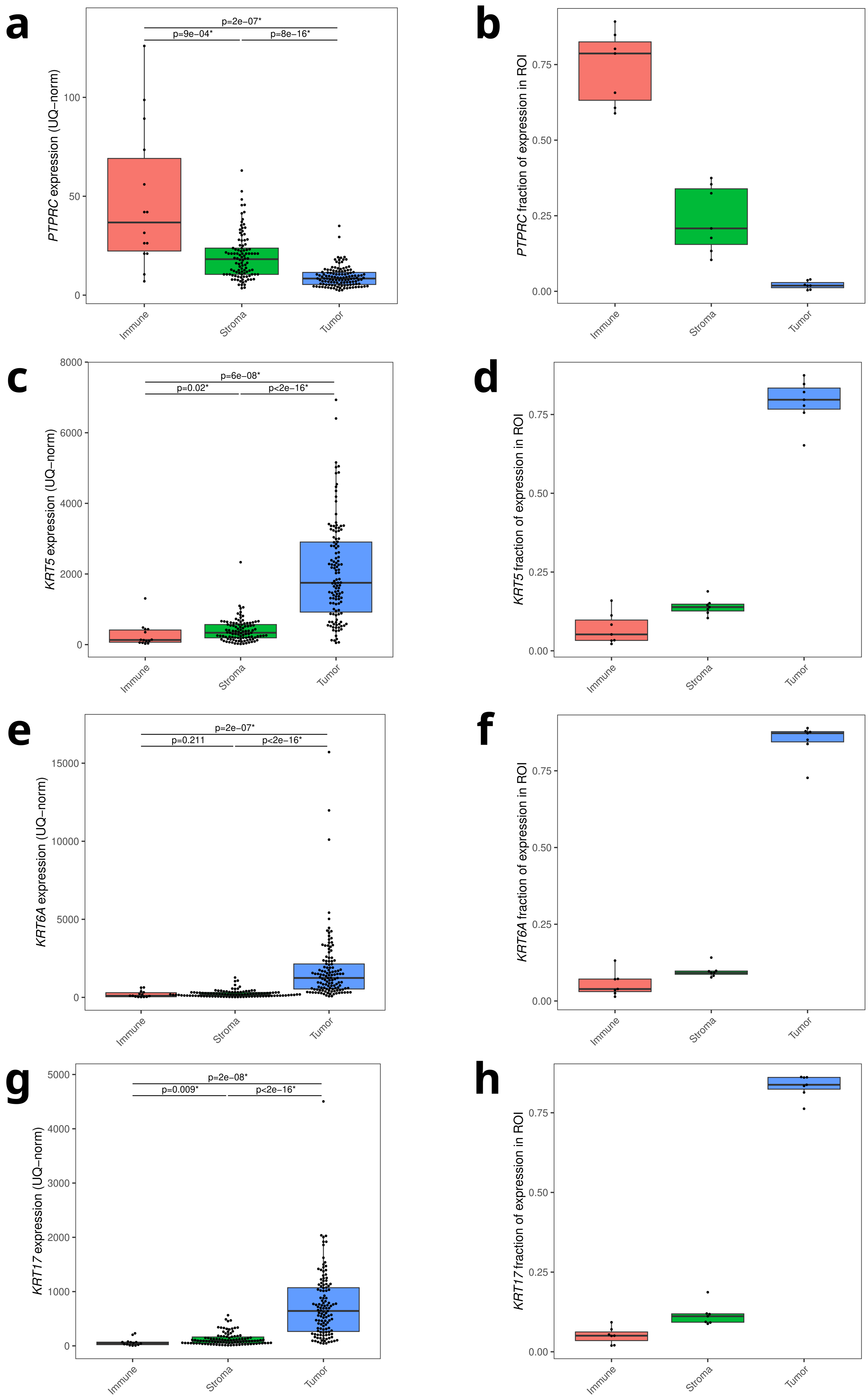

a
p=2e−07*
p=9e−04*
p=8e−16*
PTPRC expression (UQ−norm)
Immune
Stroma
Tumor
b
PTPRC fraction of expression in ROI
c
p=6e−08*
p=0.02*
p<2e−16*
KRT5 expression (UQ−norm)
d
KRT5 fraction of expression in ROI
e
p=2e−07*
p=0.211
p<2e−16*
KRT6A expression (UQ−norm)
f
KRT6A fraction of expression in ROI
g
p=2e−08*
p=0.009*
p<2e−16*
KRT17 expression (UQ−norm)
h
KRT17 fraction of expression in ROI

Fig. S2. *CD45* (*PTPRC*) and keratin transcript levels per segment. Gene expression values (UQ-normalized) are shown for immune, stroma, and tumor regions of interest (ROIs/AOIs) in **a**, **c**, **e**, and **g**. Asterisks indicate significance after multiple testing correction (FDR 5%). **b**, **d**, **f**, and **h** show the fraction of gene expression per ROI and compartment for cases with all compartments present after QC (n=6), following background subtraction. Background expression for each gene was defined as the median expression in the segment with the lowest values (tumor for *PTPRC*; immune for keratins).

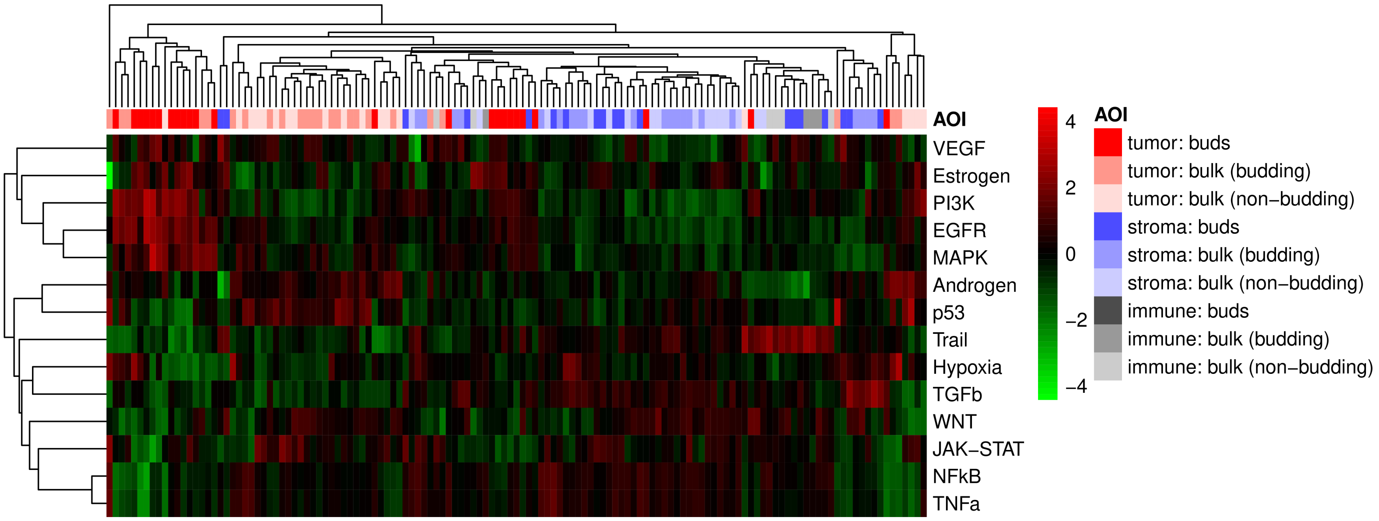

Fig. S3. Heatmap and clustering of the samples based on the PROGENy pathway activity scores.

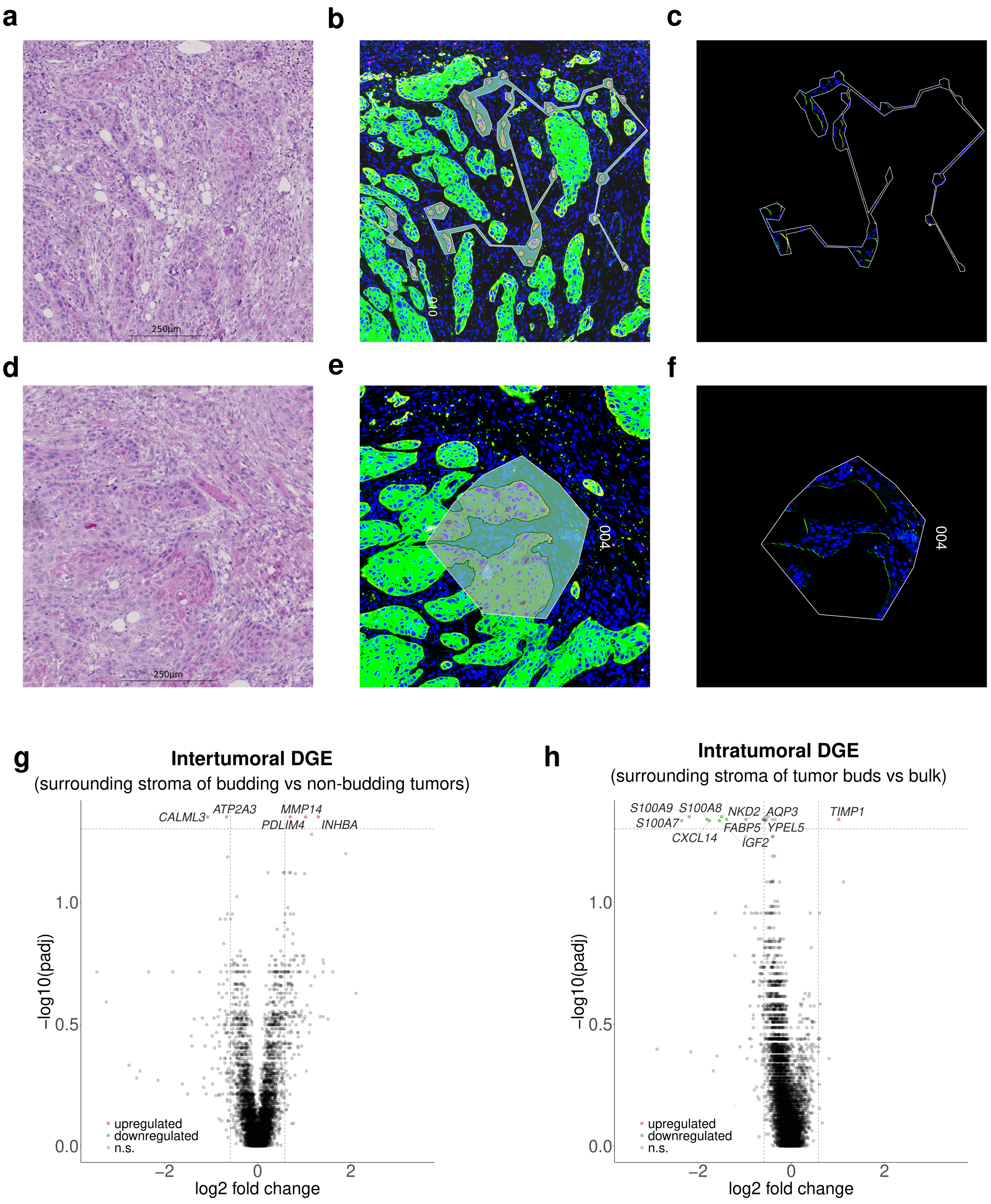

Fig. S4. Stromal differential gene expression analyses. a H&E of the budding region before tumor bud ROI selection. **b** tumor bud ROI before AOI segmentation **c** tumor bud ROI after AOI segmentation for the stroma compartment. a, b, and c represent the same region of the tumor. **d** H&E of a region before tumor bulk ROI selection. **e** tumor bulk ROI before AOI segmentation **f** tumor bulk ROI after AOI segmentation for the stroma compartment. d, e, and f represent the same region of the tumor. **g** intertumoral differential gene expression analysis (DGEA) comparing the stroma adjacent to tumor bulk of the budding against the stroma adjacent to tumor bulk of the non-budding tumors. **h** intertumoral (DGEA) comparing the stroma adjacent to tumor buds against the stroma adjacent to tumor bulk.

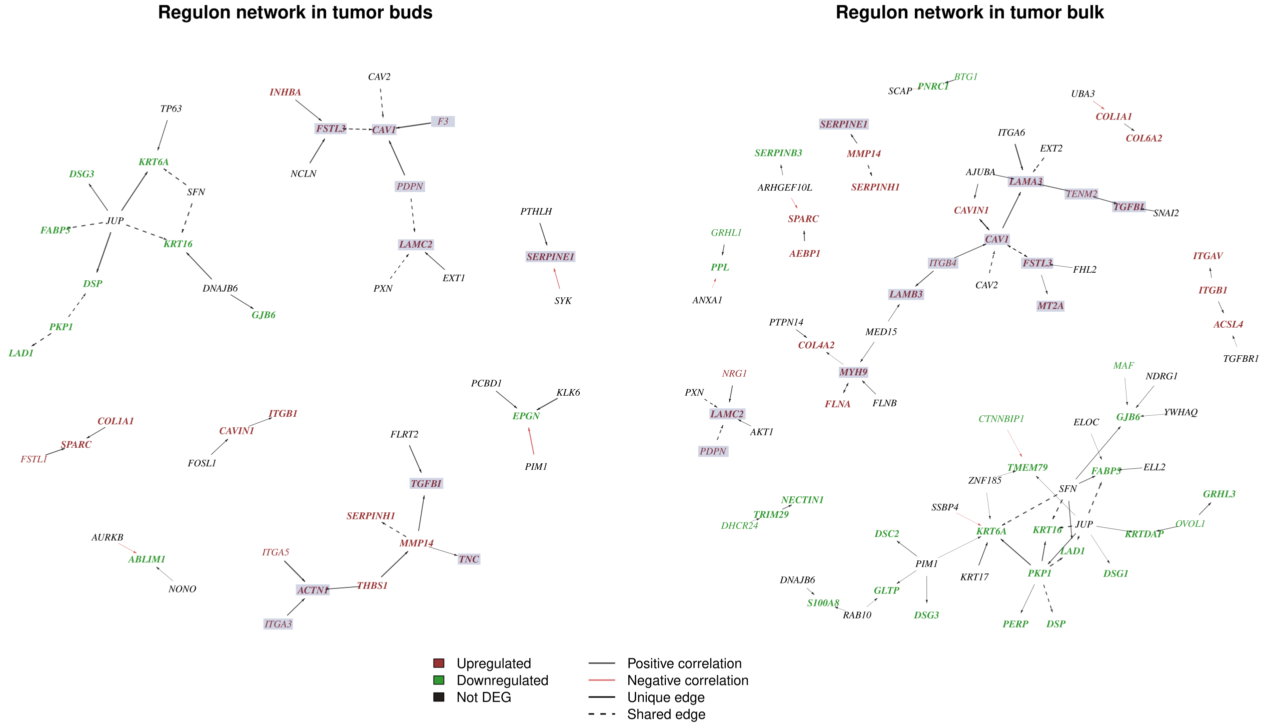

Supplementary Fig. 5. **Regulatory network of the tumor buds and corresponding tumor bulk of budding tumors based on the ARANCe-inferred TCGA-HNSC regulatory network.** The arrows show the direction of regulation (regulating gene > target gene). The line thickness corresponds to the Spearman rho correlation between the pairs. Genes in bold are members of the top 40 upregulated and 40 downregulated genes between tumor buds and tumor bulk of budding tumors. Subnetworks with only a single edge were excluded for clarity. Grey boxes mark the genes consistently upregulated in tumor buds when compared to all other compartments and segments.

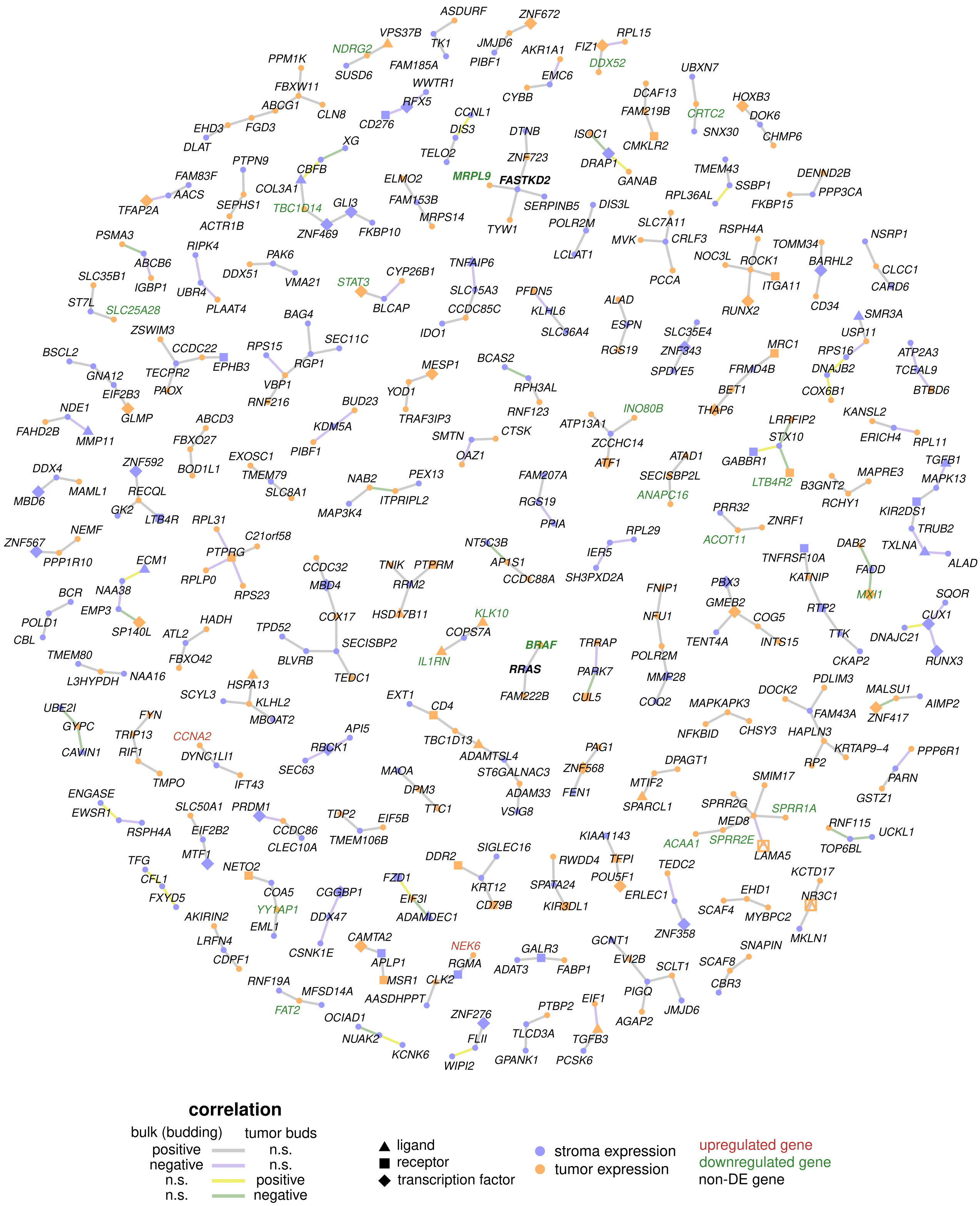
ASDURF
ZNF672
VPS37B
NDRG2
TK1
JMJD6
RPL15
FIZ1
AKR1A1
PPM1K
FAM185A
PIBF1
DDX52
SUSD6
EMC6
UBXN7
WWTR1
FBXW11
RFX5
CYBB
DCAF13
ABCG1
CD276
CLN8
CCNL1
FAM219B
CRTC2
HOXB3
EHD3
FGD3
DIS3
DTNB
ISOC1
DOK6
DLAT
XG
CMKLR2
SNX30
CHMP6
TELO2
ZNF723
DRAP1
PTPN9
CBFB
TMEM43
DENND2B
FAM83F
MRPL9
FASTKD2
GANAB
COL3A1
ELMO2
SSBP1
AACS
FAM153B
SERPINB5
RPL36AL
PPP3CA
TFAP2A
SEPHS1
TBC1D14
GLI3
DIS3L
FKBP15
ACTR1B
ZNF469
FKBP10
MRPS14
TYW1
POLR2M
SLC7A11
PSMA3
RSPH4A
RIPK4
MVK
CRLF3
TOMM34
NSRP1
ABCB6
PAK6
LCLAT1
NOC3L
SLC35B1
DDX51
ROCK1
BARHL2
CLCC1
IGBP1
UBR4
VMA21
STAT3
CYP26B1
TNFAIP6
PCCA
ITGA11
CARD6
ST7L
SLC25A28
PLAAT4
BLCAP
SLC15A3
PFDN5
ALAD
RUNX2
CD34
BAG4
CCDC85C
KLHL6
SMR3A
ZSWIM3
SEC11C
IDO1
SLC36A4
ESPN
SLC35E4
USP11
CCDC22
RPS15
RGS19
ZNF343
MRC1
RPS16
ATP2A3
BSCL2
EPHB3
RGP1
BCAS2
SPDYE5
FRMD4B
DNAJB2
TCEAL9
GNA12
TECPR2
MESP1
EIF2B3
PAOX
VBP1
YOD1
RPH3AL
BET1
COX6B1
BTBD6
BUD23
RNF216
NDE1
INO80B
GLMP
TRAF3IP3
RNF123
THAP6
KANSL2
ABCD3
ATP13A1
LRRFIP2
FAHD2B
MMP11
KDM5A
SMTN
CTSK
ERICH4
FBXO27
STX10
RPL11
PIBF1
ZCCHC14
ATAD1
EXOSC1
OAZ1
GABBR1
TGFB1
DDX4
ZNF592
BOD1L1
ATF1
MAPRE3
TMEM79
FAM207A
SECISBP2L
LTB4R2
B3GNT2
MAPK13
MBD6
MAML1
NAB2
PEX13
RECQL
SLC8A1
ANAPC16
RCHY1
GK2
ITPRIPL2
RGS19
KIR2DS1
LTB4R
MAP3K4
PRR32
ZNRF1
RPL31
PPIA
TRUB2
NEMF
C21orf58
ACOT11
RPL29
DAB2
TXLNA
ZNF567
PTPRG
NT5C3B
IER5
ALAD
PPP1R10
ECM1
TNIK
PTPRM
AP1S1
TNFRSF10A
FADD
CCDC32
RPLP0
RRM2
CCDC88A
SH3PXD2A
KATNIP
NAA38
MBD4
PBX3
BCR
RPS23
FNIP1
MXI1
SQOR
GMEB2
RTP2
EMP3
KLK10
CUX1
POLD1
HSD17B11
COX17
NFU1
COG5
COPS7A
DNAJC21
SP140L
HADH
TTK
CBL
TENT4A
TPD52
SECISBP2
BRAF
TRRAP
INTS15
ATL2
TMEM80
BLVRB
POLR2M
IL1RN
RUNX3
CKAP2
FBXO42
RRAS
NAA16
PARK7
L3HYPDH
TEDC1
MMP28
HSPA13
PDLIM3
SCYL3
DOCK2
UBE2I
MALSU1
FAM222B
COQ2
CUL5
EXT1
AIMP2
KLHL2
CD4
MAPKAPK3
FAM43A
FYN
GYPC
MBOAT2
ZNF417
NFKBID
CHSY3
HAPLN3
TBC1D13
API5
TRIP13
CCNA2
KRTAP9−4
CAVIN1
RIF1
ADAMTSL4
PAG1
PPP6R1
RBCK1
DYNC1LI1
RP2
DPAGT1
ST6GALNAC3
ZNF568
SEC63
MAOA
SMIM17
PARN
TMPO
IFT43
ADAM33
MTIF2
DPM3
FEN1
GSTZ1
ENGASE
SPRR2G
SLC50A1
PRDM1
SPARCL1
EWSR1
TTC1
SPRR1A
VSIG8
TDP2
MED8
RNF115
EIF5B
UCKL1
RSPH4A
EIF2B2
CCDC86
ACAA1
KIAA1143
SPRR2E
TMEM106B
TOP6BL
CLEC10A
MTF1
SIGLEC16
LAMA5
TFG
RWDD4
DDR2
TFPI
TEDC2
NETO2
CFL1
KCTD17
POU5F1
FZD1
KRT12
EHD1
SPATA24
FXYD5
COA5
CGGBP1
ERLEC1
NR3C1
EIF3I
CD79B
YY1AP1
KIR3DL1
SCAF4
AKIRIN2
MYBPC2
ADAMDEC1
DDX47
ZNF358
EML1
LRFN4
CAMTA2
MKLN1
GCNT1
NEK6
CSNK1E
SNAPIN
GALR3
CDPF1
EVI2B
APLP1
RGMA
ADAT3
FABP1
SCAF8
RNF19A
MSR1
CLK2
SCLT1
MFSD14A
PIGQ
CBR3
AASDHPPT
JMJD6
FAT2
EIF1
PTBP2
ZNF276
OCIAD1
AGAP2
NUAK2
TLCD3A
FLII
TGFB3
KCNK6
GPANK1
PCSK6
WIPI2
correlation
bulk (budding)
tumor buds
positive
n.s.
negative
n.s.
n.s.
positive
n.s.
negative
ligand
receptor
transcription factor
stroma expression
tumor expression
upregulated gene
downregulated gene
non-DE gene

Fig. S6. **Differential correlation of gene expression between tumor buds and bulk.** Networks with more than 2 and fewer than 8 members. Each node refers to a gene, with tumoral (orange) and stromal (purple) expression shown separately. The shape of the node refers to a functional annotation (ligand, receptor, transcription factor) and gene pairs in bold font refer to known interactions. The edges connect two genes when their correlation of expression was differential, with different colors showing the direction of correlation change between tumor buds and bulk.

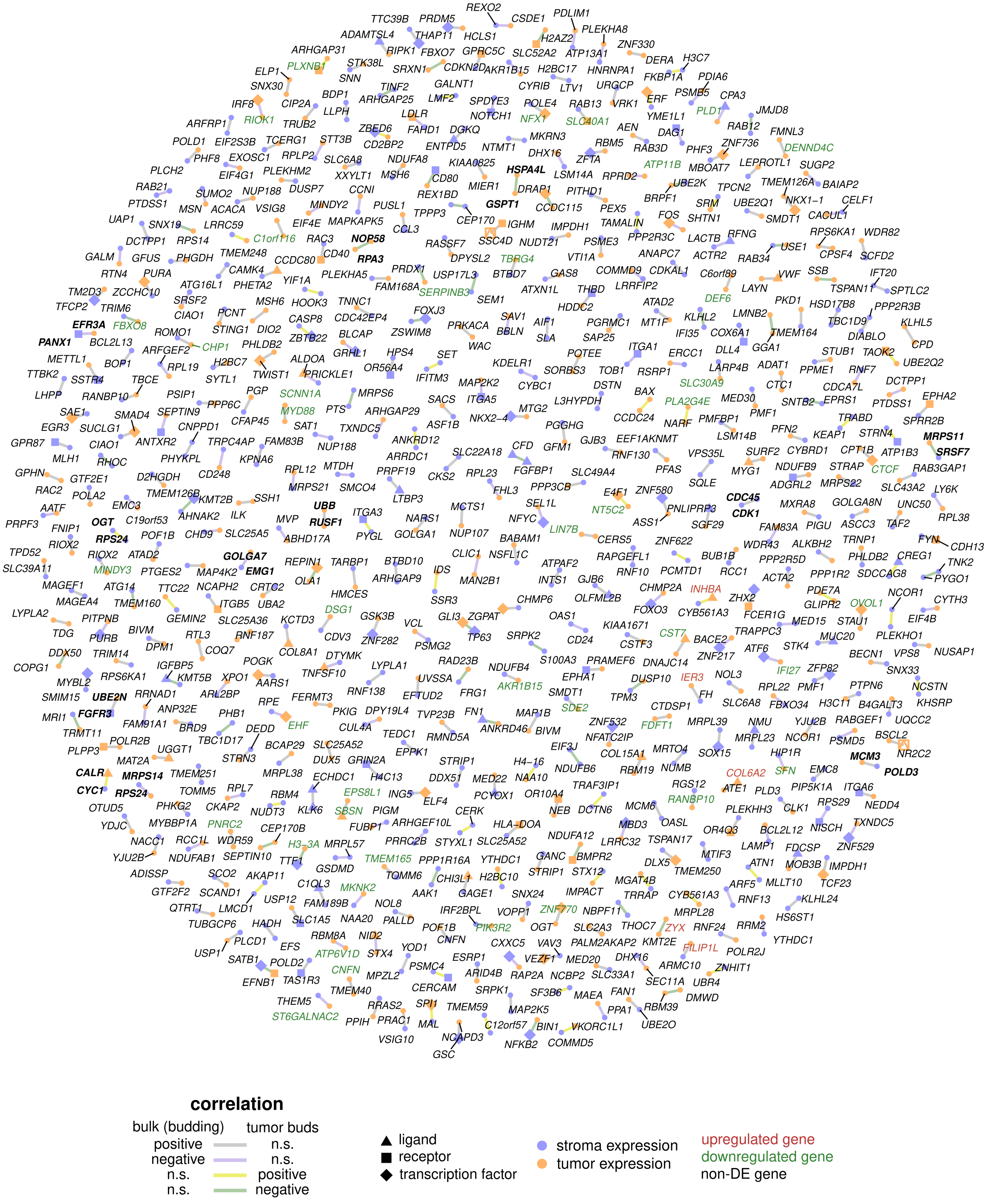

correlation
bulk (budding)
tumor buds
positive
n.s.
negative
n.s.
n.s.
positive
n.s.
negative
ligand
receptor
transcription factor
stroma expression
tumor expression
upregulated gene
downregulated gene
non-DE gene

Fig. S7. **Differential correlation of gene expression between tumor buds and bulk.** Networks with 2 members. Each node refers to a gene, with tumoral (orange) and stromal (purple) expression shown separately. The shape of the node refers to a functional annotation (ligand, receptor, transcription factor) and gene pairs in bold font refer to known interactions. The edges connect two genes when their correlation of expression was differential, with different colors showing the direction of correlation change between tumor buds and bulk.

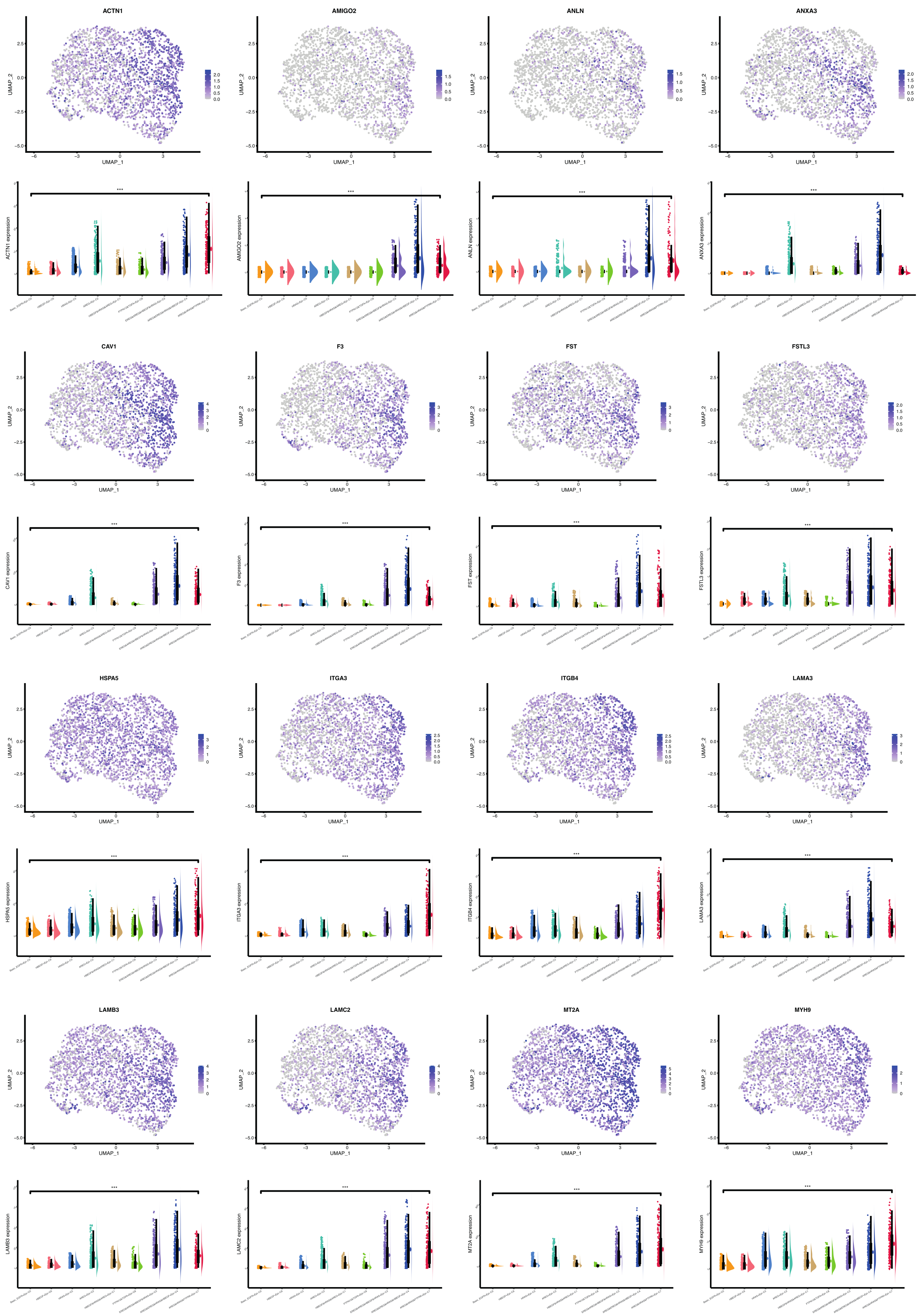
ACTN1
AMIGO2
ANLN
ANXA3
UMAP_1
UMAP_2
ACTN1 expression
AMIGO2 expression
ANLN expression
ANXA3 expression
CAV1
F3
FST
FSTL3
CAV1 expression
F3 expression
FST expression
FSTL3 expression
HSPA5
ITGA3
ITGB4
LAMA3
HSPA5 expression
ITGA3 expression
ITGB4 expression
LAMA3 expression
LAMB3
LAMC2
MT2A
MYH9
LAMB3 expression
LAMC2 expression
MT2A expression
MYH9 expression

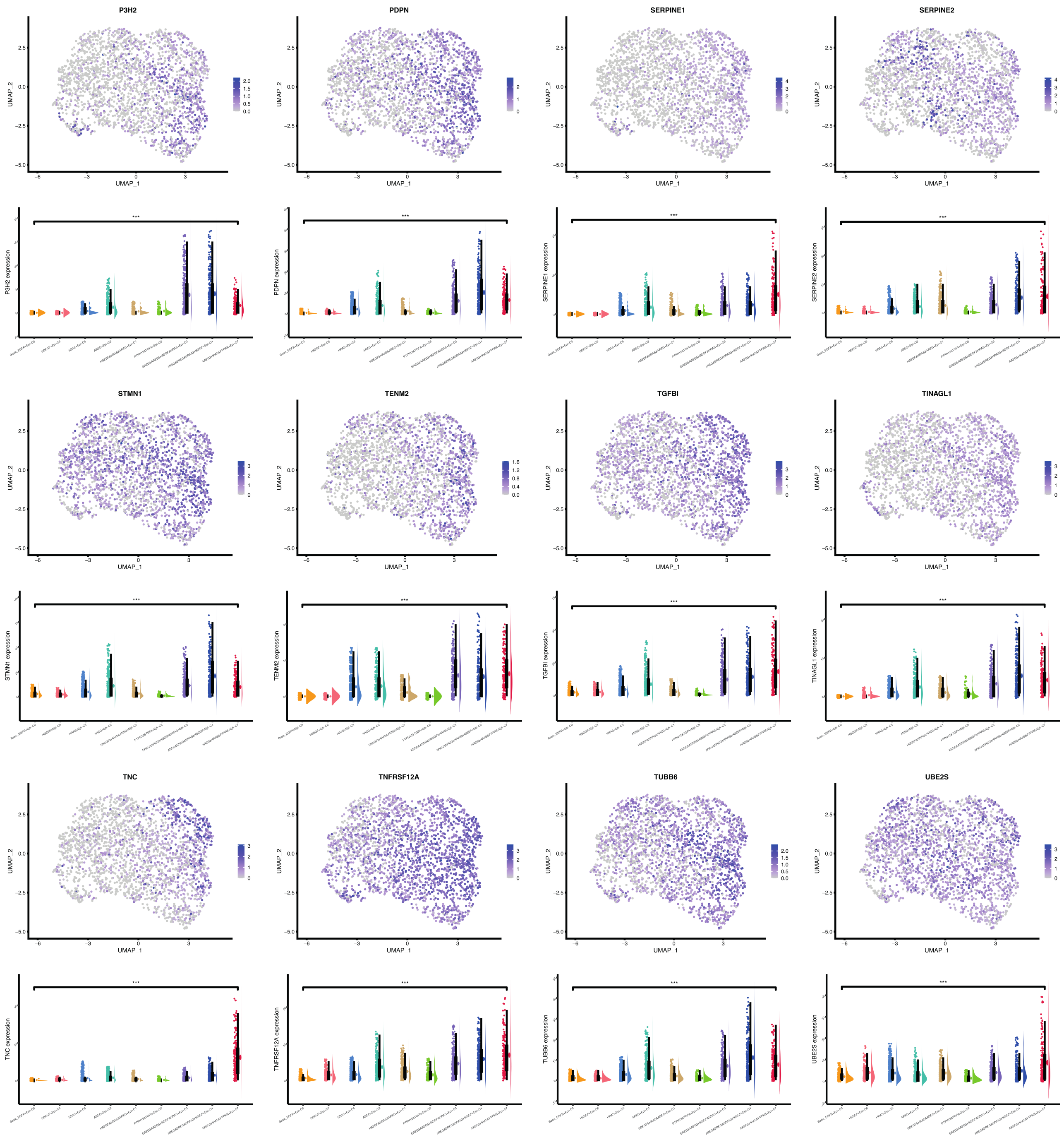

Fig. S8. TBS genes and EGFR subtypes. The expression of the indicated n = 28 genes composing the tumor budding biomarker score (TBS) is presented in uMAPs of single malignant cells of GSE181919 together with rainbow plots of expression strength of each gene in EGFR-activity subtypes.

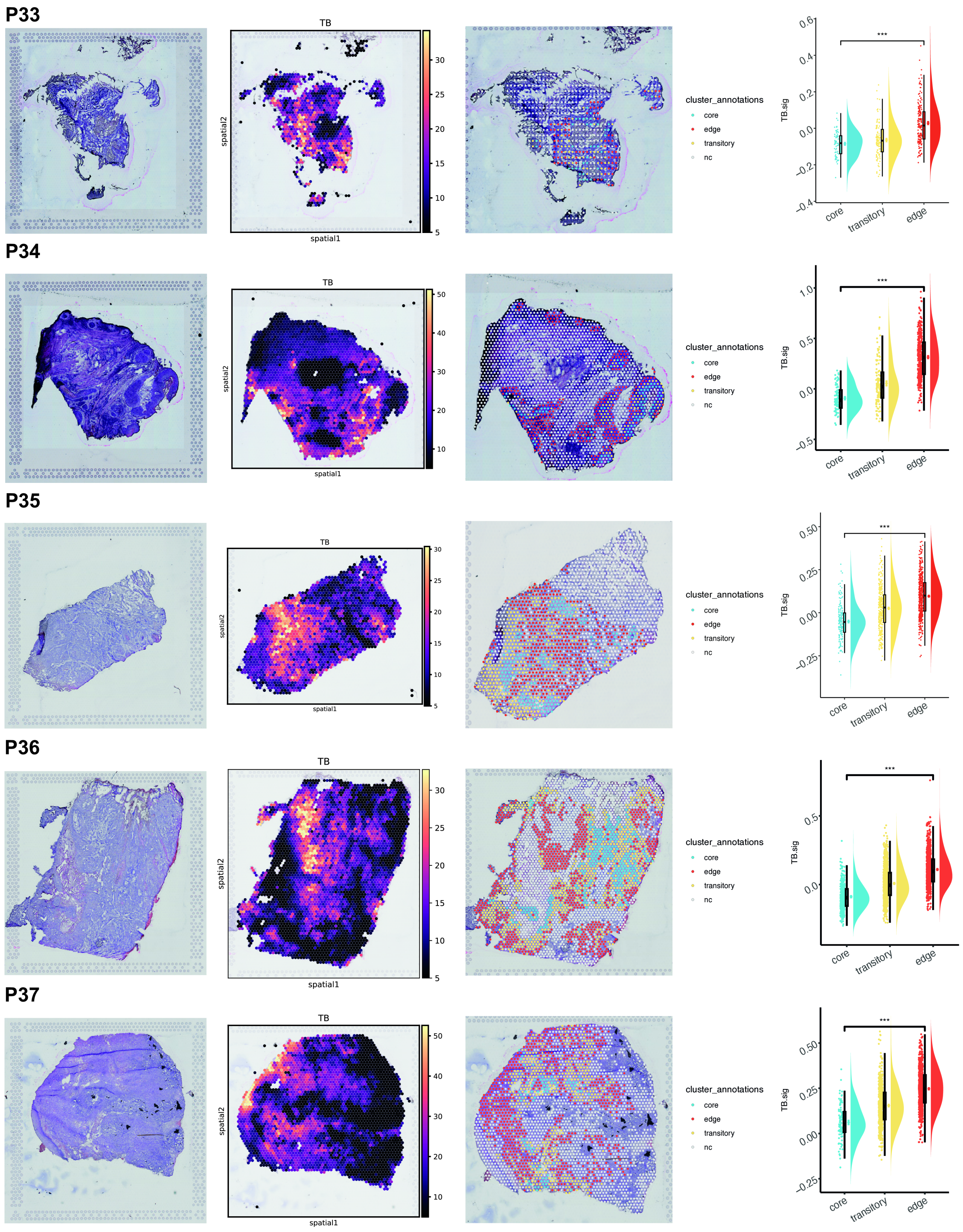
P33
TB
spatial1
spatial2
5
10
15
20
25
30
cluster_annotations
core
edge
transitory
nc
TB.sig
***
0.6
0.4
0.2
0.0
-0.2
-0.4
P34
TB
spatial1
spatial2
10
20
30
40
50
cluster_annotations
core
edge
transitory
nc
TB.sig
***
1.0
0.5
0.0
-0.5
P35
TB
spatial1
spatial2
5
10
15
20
25
30
cluster_annotations
core
edge
transitory
nc
TB.sig
***
0.50
0.25
0.00
-0.25
P36
TB
spatial1
spatial2
5
10
15
20
25
30
cluster_annotations
core
edge
transitory
nc
TB.sig
***
0.5
0.0
P37
TB
spatial1
spatial2
10
20
30
40
50
cluster_annotations
core
edge
transitory
nc
TB.sig
***
0.50
0.25
0.00
-0.25
core
transitory
edge

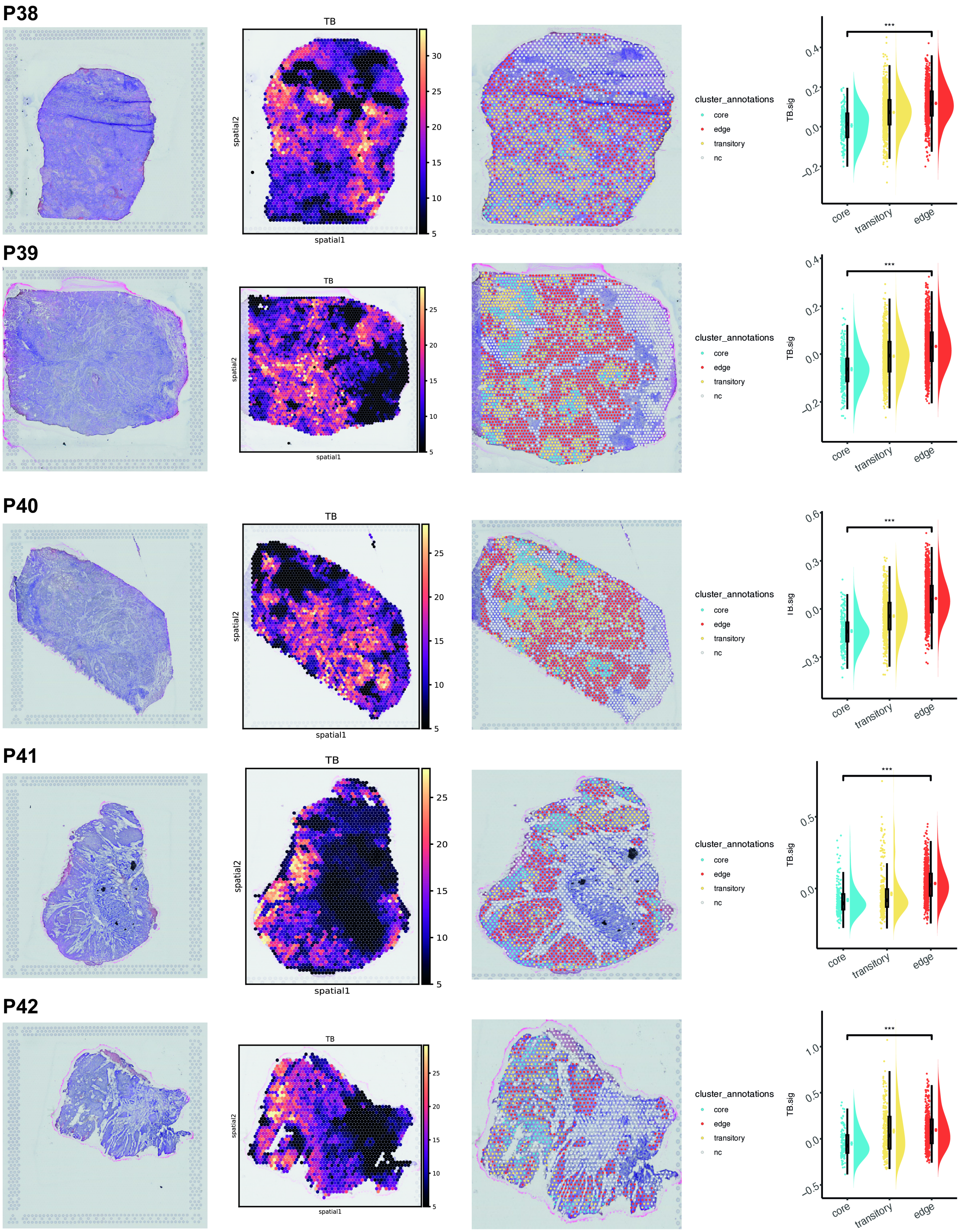

P38
TB
spatial2
spatial1
30
25
20
15
10
5
cluster_annotations
core
edge
transitory
nc
TB.sig
***
0.4
0.2
0.0
-0.2
core
transitory
edge
P39
TB
spatial2
spatial1
25
20
15
10
5
cluster_annotations
core
edge
transitory
nc
TB.sig
***
0.4
0.2
0.0
-0.2
core
transitory
edge
P40
TB
spatial2
spatial1
25
20
15
10
5
cluster_annotations
core
edge
transitory
nc
TB.sig
***
0.6
0.3
0.0
-0.3
core
transitory
edge
P41
TB
spatial2
spatial1
25
20
15
10
5
cluster_annotations
core
edge
transitory
nc
TB.sig
***
0.5
0.0
core
transitory
edge
P42
TB
spatial2
spatial1
25
20
15
10
5
cluster_annotations
core
edge
transitory
nc
TB.sig
***
1.0
0.5
0.0
-0.5
core
transitory
edge

Fig. S9. TBS spatial expression. The spatial distribution of the tumor budding biomarker score (TBS) was assessed in spatial transcriptomic dataset GSE208253. Shown are H&E staining, TBS distribution and strength, core, edge, and transitory regions for each indicated patient. TBS was calculated for core, edge, and transitory regions, and is represented for each patient in a rainbow plot.

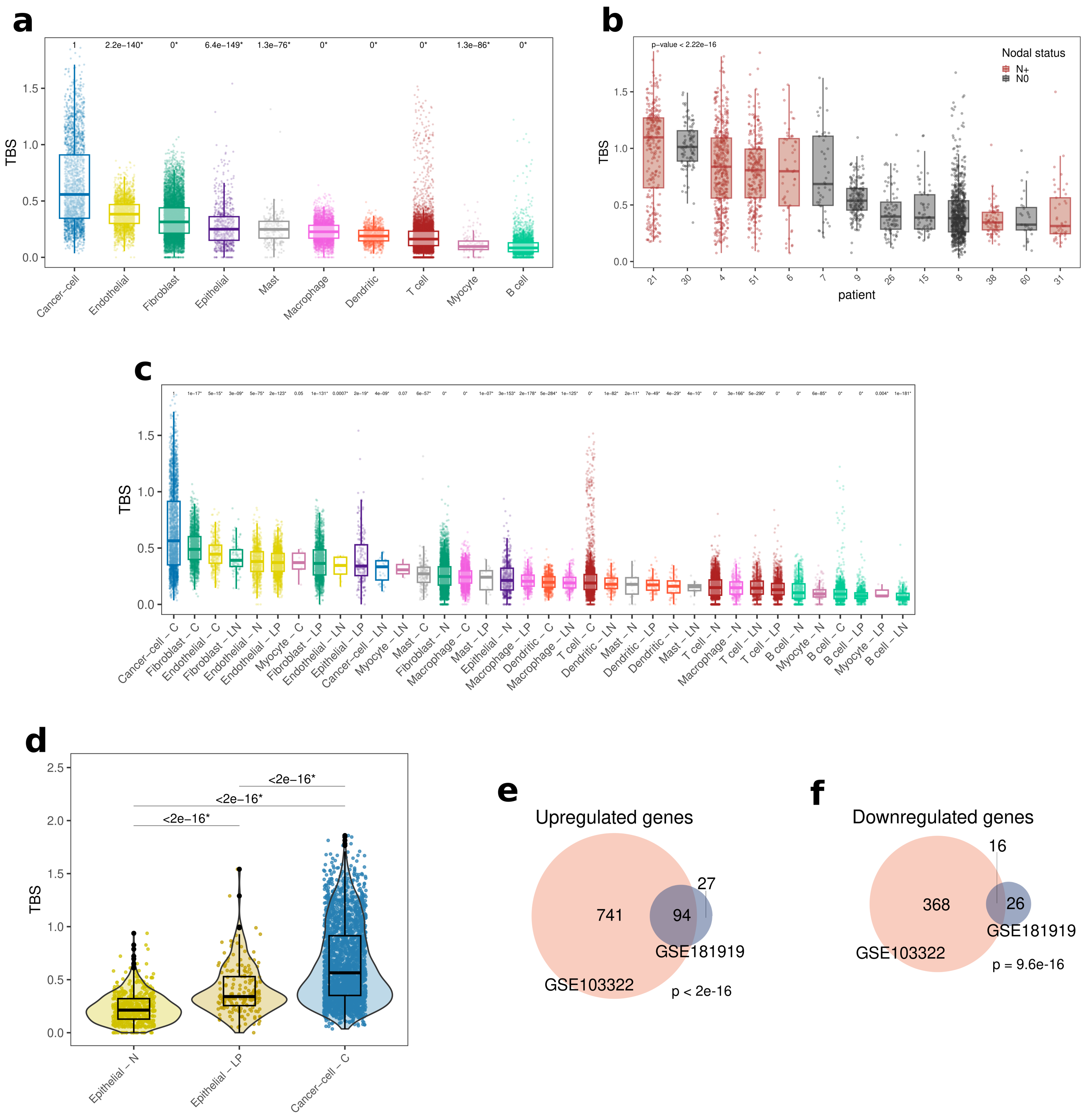

Fig. S10. scRNA-seq (GSE181919) and tumor budding signature (TBS). **a** TBS by cell type. **b** TBS of primary cancer cells by patient. **c** TBS by cell type and sample type (N: normal sample; LP: leukoplakia sample; C: primary tumor sample; LN: lymph node sample). **d** Comparison of the TBS score between the epithelial cells of the normal samples, the epithelial cells of the leukoplakia samples and the cancer cells of the primary tumor samples. The asterisks indicate significance after multiple testing correction. **e-f** Overlap of the GSE181919 and GSE103322 scRNA-seq differentially expressed genes (DEGs) between the TBS-high and TBS-low primary cancer cells. Upregulated (e) and downregulated (f) DEGs are shown separately.

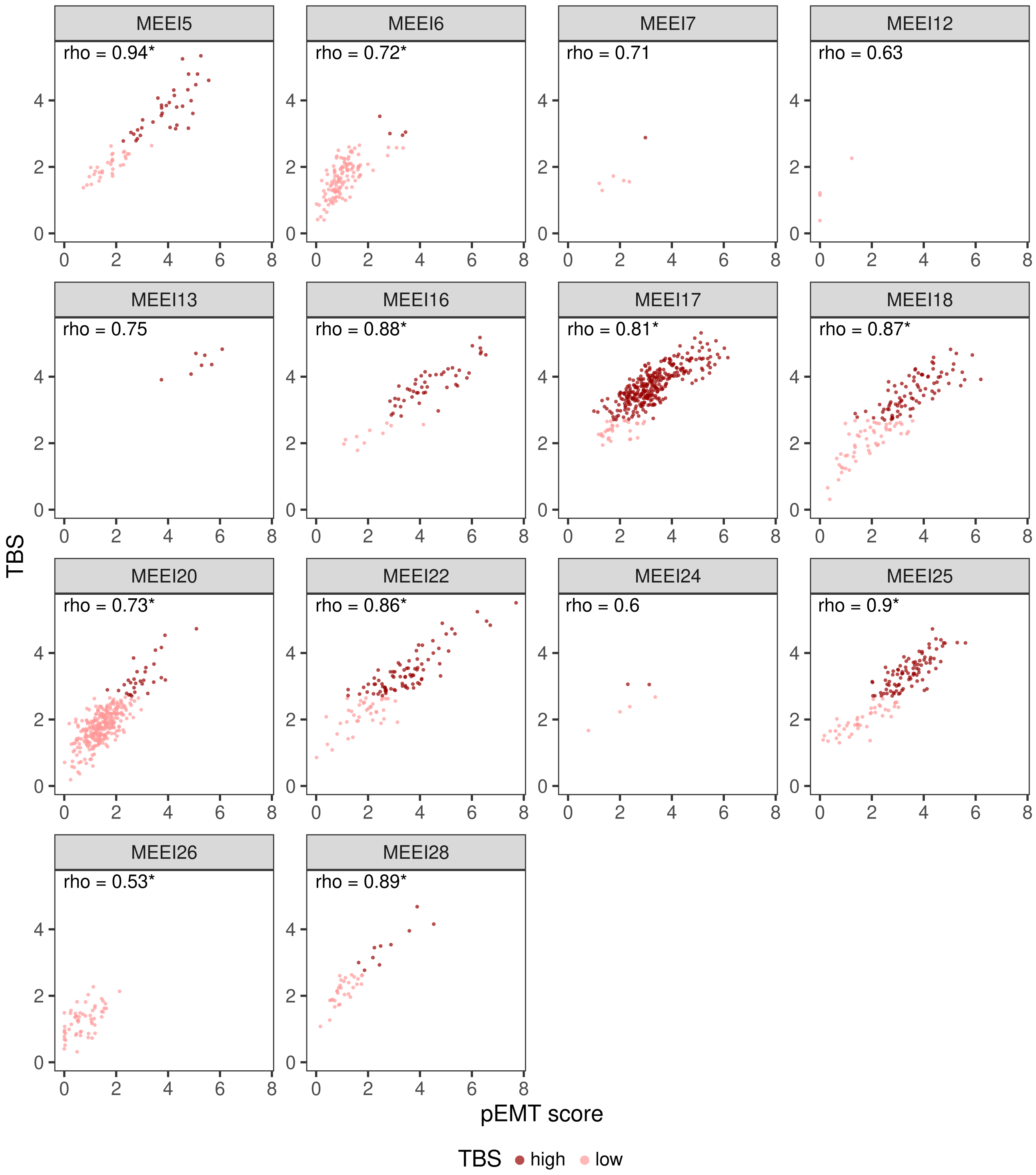

Fig. S11. TBS and pEMT score correlation of the primary tumor cells per patient. Patients at least two primary cancer cells sequenced are shown. Asterisks indicate significance after multiple testing correction (at FDR 5%).
